# Inhibition of anti-tumor immunity by melanoma cell-derived Activin-A depends on STING

**DOI:** 10.3389/fimmu.2023.1335207

**Published:** 2024-01-18

**Authors:** Katarina Pinjusic, Giovanna Ambrosini, Joao Lourenco, Nadine Fournier, Christian Iseli, Nicolas Guex, Olga Egorova, Sina Nassiri, Daniel B. Constam

**Affiliations:** ^1^ Ecole Polytechnique Fédérale de Lausanne (EPFL), SV ISREC, Lausanne, Switzerland; ^2^ Bioinformatics Competence Center, Ecole Polytechnique Fédérale de Lausanne, Lausanne, Switzerland; ^3^ Bioinformatics Competence Center, Université de Lausanne, Lausanne, Switzerland; ^4^ Translational Data Science Facility, Swiss Institute of Bioinformatics, AGORA Cancer Research Center, Lausanne, Switzerland

**Keywords:** cancer, intercellular communication, scRNA-seq, profiling, knockdown, activin, interferon, STING

## Abstract

The transforming growth factor-β (TGF-β) family member activin A (hereafter Activin-A) is overexpressed in many cancer types, often correlating with cancer-associated cachexia and poor prognosis. Activin-A secretion by melanoma cells indirectly impedes CD8^+^ T cell-mediated anti-tumor immunity and promotes resistance to immunotherapies, even though Activin-A can be proinflammatory in other contexts. To identify underlying mechanisms, we here analyzed the effect of Activin-A on syngeneic grafts of *Braf* mutant YUMM3.3 mouse melanoma cells and on their microenvironment using single-cell RNA sequencing. We found that the Activin-A-induced immune evasion was accompanied by a proinflammatory interferon signature across multiple cell types, and that the associated increase in tumor growth depended at least in part on pernicious STING activity within the melanoma cells. Besides corroborating a role for proinflammatory signals in facilitating immune evasion, our results suggest that STING holds considerable potential as a therapeutic target to mitigate tumor-promoting Activin-A signaling at least in melanoma.

## Introduction

Therapies that enhance the ability of the immune system to recognize and eliminate cancer cells have significantly improved patient survival across some but not all tumor types (1). Ongoing clinical efforts to boost anti-tumor immunity include targeted delivery of agonists of the stimulator of interferon response cGAMP interactor (STING) pathway (2). However, chronic activation of this and other pro-inflammatory signals such as interferon (IFN)-γ can lead to tolerogenic responses and immune evasion (2–5). The efficacy or durability of available immunotherapies thus is limited by primary or acquired therapy resistance in a large proportion of patients. Mechanisms of resistance include the paucity of tumor antigens or their inefficient presentation to immune cells, resistance to T cell-mediated killing, and T cell exclusion that can be mediated by immunosuppressive cells in the tumor microenvironment (TME), inhibition of dendritic cell (DC) maturation or recruitment, downregulation of proinflammatory cytokines, or upregulation of immune checkpoint receptors and their ligands or other inhibitory factors (3, 4). In addition, chronic inflammation of tumors evading immune-mediated elimination frequently reprograms the microenvironment to provide a panoply of factors that stimulate tumor vascularization and cancer cell survival, proliferation, and migration, thereby promoting disease progression instead of tumor immune surveillance.

Activin-A, a secreted TGF-β related protein encoded by the *INHBA* gene, binds to complexes of cognate activin receptors (ActR)-IIA or -IIB with ActR-IB (also known as ALK4), or with the lower affinity type I receptor ActR-IC (also known as ALK7 or ACVR1C) (6). These proteins are encoded by *ACVR2A*, *ACVR2B*, and *ACVR1B or ACVR1C*, respectively. Receptor binding enables the phosphorylation and nuclear translocation of the transcription factors SMAD2 and SMAD3, as well as non-canonical signal transduction mediated by phosphoinositide 3-kinase (PI3K) and mitogen-activated kinase (MAPK) pathways (7, 8). Activin-A can be secreted by numerous cell types, including cells of the innate and adaptive immune systems, where it can promote or inhibit immune responses, depending on the cell state (9). Activin-A signaling can also inhibit or promote tumor progression, depending on the tumor type and its context (10–12). Elucidating tumor-promoting functions of *INHBA* and their underlying mechanisms is important, because increased expression and elevated circulating Activin-A levels in various cancer types correlate with poor prognosis and with cancer-associated cachexia (13–15). In addition, several recent studies implicate Activin-A in facilitating tumor immune evasion and immunotherapy resistance. In particular, overexpression of *INHBA* in syngeneic grafts of mouse B16-F1 or YUMM3.3 mouse melanoma, or inhibition of endogenous Activin-A in syngeneic iBIP2 melanoma grafts by a ligand trap revealed that Activin-A secretion by the cancer cells stimulates both primary and metastatic tumor growth specifically in immunocompetent hosts, but not in *nu/nu* or *Rag1* ^-/-^ mice lacking adaptive immunity (16, 17). Immune profiling of these preclinical melanoma models by flow cytometry, together with T cell depletion experiments and adoptive cell transfers showed that Activin-A inhibits anti-tumor immunity by attenuating CD8^+^ T cell infiltration, correlating with diminished secretion of the chemokines CXCL9 and CXCL10 by myeloid cells. By contrast, the activation of cytotoxic T cells by antigen *in vitro* and their cytotoxicity after adoptive cell transfer were not inhibited, confirming that Activin-A impaired their function indirectly (17). Immunosuppressive Activin-A signaling in melanoma and possibly other cancers is likely clinically relevant because elevated *INHBA* expression correlates with resistance to immune checkpoint blockade (ICB) therapy in melanoma patients and impairs the response to both ICB and T cell-based immunotherapy in mouse melanoma grafts (17). In keeping with a role in promoting tumor immune evasion, *INHBA* overexpression in keratinocytes in a transgenic model of skin squamous cell carcinoma has been shown to alter the TME by enriching immunosuppressive macrophages and regulatory T cells at the expense of skin-resident γδ T cells (18, 19). In addition, recent studies using single-cell and spatial transcriptomics analysis of basal cell carcinomas have identified Activin-A as a tissue remodeling factor in the invasive niche and as a biomarker for an immunosuppressive TME with CD8 T cell exclusion in ICB-resistant patients (20, 21). Together, these findings established Activin-A as an important TME remodeling factor that promotes tumor immune evasion and resistance to ICB therapy. However, a comprehensive analysis of the TME composition and transcriptome changes induced by Activin-A in cancer is lacking.

Here, we used the syngeneic mouse melanoma model YUMM3.3 and single-cell RNA sequencing (scRNA-seq) analysis to interrogate the changes induced by *INHBA* overexpression in tumor cells. Besides identifying cell types in the TME that respond to Activin-A, our analysis shows that Activin-A-induced melanoma growth involves the STING pathway and upregulation of interferon (IFN) signaling via JAK transcription factors. Together, these findings shed important new light on the mechanisms mediating a tumor-promoting function of Activin-A in melanoma.

## Materials and methods

### Cell lines

For all cell lines, culture media were supplemented with 10% fetal bovine serum, 50 µg/mL gentamicin (Gibco, Thermo Fisher Scientific, Waltham, MA, USA), and 1% GlutaMAX (Gibco). HEK293T cells were purchased from ATCC and maintained in DMEM (Sigma-Aldrich, St. Louis, MO, USA). YUMM3.3-Ctrl and -βA cells were previously described (17) and maintained in DMEM/F12 (Gibco). Cell morphology was regularly inspected, and cultures tested negative for mycoplasma (Mycospy kit, Biontex) were used throughout the study.

### Lentiviral transduction

YUMM3.3-Ctrl and -βA cells with stable expression of GFP were generated by lentiviral transduction. In short, HEK293T cells were co-transfected with CMVΔR8.74 (Addgene, Watertown, MA, USA 22036), pMD2.VSVg (Addgene 12259) and GFP containing transfer vector, shLuc, shSting 320 or shSting 266 transfer plasmid (provided by Dr. Denarda Dangaj Laniti) or Fucci reporter (provided by Dr. Cathrin Brisken). Lentiviral particles were collected from filtered culture supernatant by ultracentrifugation and resuspended in sterile PBS. YUMM3.3-Ctrl or -βA cells were transduced in a 12-well plate. GFP transduction efficiency was over 95% and was not further selected, while shLuc,shSting 320 and 266 cell lines were selected by culturing for 2 weeks with 300 µg/ml G418 (InvivoGen, ant-gn).

### Cell viability assay

For quantification of the cytostatic effect of IFN-γ, YUMM3.3 cells were seeded at a density of 5x10^3^ cells/well in 96-well plate and incubated 48 hrs with 20 ng/ml IFN-γ (485-MI, R&D) and 50 ng/ml Activin-A (R&D Systems) or 10 μM SB-431542, and, where indicated, Ruxolitinib or Fludarabine, 0.5 μM Nifuroxazide (all from MedChem Express), 100 μg/ml Carboplatin (C2043-1G, TCI America), E64 (HY-15282, MedChemExpress), or Petesicatib (HY-109069, MedChemExpress). Alamar Blue reagent (Invitrogen, DAL1025) was added to subconfluent cells (<90% confluence, inspected manually), and fluorescence was measured 3-4 hrs later on a TECAN spectrophotometer at the emission wavelength of 590 nm after excitation at 560 nm.

### Western blot analysis

2x10^5^ YUMM3.3 cells were seeded in 24-well plates and incubated with 20 ng/ml IFN-γ (485-MI, R&D Systems), 10 μM SB-431542, 50 ng/ml of Activin-A for 24 h where indicated. Cells were lysed in RIPA buffer supplemented with protease inhibitor cocktail (Roche, Basel, CH) and phosphatase inhibitors (Sigma-Aldrich). Proteins were separated on 9-12% SDS-PAGE gels under reducing conditions and transferred on nitrocellulose membranes. Membranes were blocked with 5% skim milk (Sigma) in Tris-buffered saline containing 0.1% Tween-20, before incubation with primary antibodies against γ-tubulin (Sigma GTU88) or pSTAT (9167S), STAT1 (9172), or STING (13647S) antibodies (all from Cell Signaling) for 2 hrs at room temperature or overnight at 4°C. Chemiluminescence was revealed on X-ray film (Kodak, Rochester, NY, USA) or ChemiDoc MP (Biorad, Hercules, CA, USA) using HRP-coupled secondary antibodies and ECL reagents (Thermo Fisher).

### Mouse cDC1 cell line activation and flow cytometry staining

Immortalized mouse cDC1 cells (22) were cultured in IMDM medium supplemented with 10% heat-inactivated FBS, 100x Glutamax, 10 mM HEPES, 100 μM Pen/Strep, 50 μM β-mercaptoethanol at 37° and 5% CO2. PBS supplemented with 20 mM HEPES and 5 mM EDTA was used for cell passaging. For activation, 2.5 x 10^5^ cells were seeded per well in a 12-well plate and incubated for 24 hrs with 5 μg/ml LPS and 10 ng/ml IFN-γ (485-MI, R&D Systems). Where indicated, cells were also incubated with 50 ng/ml recombinant Activin-A, TGF-β (Invitrogen), or BMP4 (Biotechne), or with 10 μM SB-431542. During the last 3 hrs of incubation, Golgi Plug (BD 555029) was added to the cells. Cells were then detached using PBS supplemented with 1% FBS and 2 mM EDTA, washed and stained for surface markers H2Kb FITC (Biolegend, clone AF88.5) and IA/IE AlexaFluor700 (BioLegend, clone M5/114.15.2), followed by fixation and permeabilization using FoxP3 staining buffer set (eBioscience) before intracellular staining with Ki67 eFluor 450 (Invitrogen, clone SolA15) and CXCL9 AlexaFluor647 (eBioscience, clone MIG-2F5.5). After the staining, cells were collected in FACS buffer (2% FBS, 2 mM EDTA in PBS), and data were acquired using an LSRII SORP or an LSR Fortessa cytometer (Becton Dickinson, Franklin Lakes, NJ, USA).

### Melanoma grafts and ruxolitinib injections

2.5x10^5^ YUMM3.3 cells were injected subcutaneously into the right flank of 8-12 week old female C57BL/6 mice (Charles River laboratories). To inhibit JAK1/2 signaling, mice were treated with 1 mg ruxolitinib (INCB018424) dissolved in 50 µl of DMSO, or a vehicle daily, starting one day before the tumor injection. Tumors were measured 3 times/week, and volumes were calculated using the formula V = [1.58π x (length x width)3/2]/6 (23). All procedures were according to Swiss legislation and approved by the cantonal veterinary administration.

### Tumor dissociation and single cell RNA sequencing

YUMM3.3-Ctrl.GFP and -βA.GFP tumors were dissected, minced using rounded scissors, and digested in Dnase-I (0.02 mg/mL, Sigma) and collagenase (1 mg/mL, Sigma) in RPMI using a gentleMACS Octo Dissociator (Miltenyi). In total, we collected six Ctrl and six βA tumors. Two tumors of each genotype were pooled for each biological replicate based on tumor volumes to have comparable starting amounts for each replicate. To minimize the time that cells spent on the ice, samples were randomized, and live single-cell sorted on two machines (Becton Dickinson FACSAria II and ACSAria Fusion) into GFP^hi^ tumor cells and GFP^int^ stromal populations. For dead cell staining, Propidium Iodide Solution (421301, Biolegend) was added to the cells immediately before sorting. Immediately after sorting, cells were counted, centrifuged at 300 g for 5 min at RT, and resuspended at a density of 1000 cells/μl in DMEM supplemented with 10% filtered FBS. GFP^hi^ fractions were added back to GFP^int^ cells to account for 10% of the final volume. Cell concentrations and quality were again assessed using trypan blue staining. Samples contained less than 5% dead cells and 3-5% doublets. Single-cell RNA sequences were obtained using 10x Genomics Chromium v3.1 kit and Illumina Hiseq 4000.

Following demultiplexing of sequencing libraries into individual FASTQ files, sequencing reads were quantified using Cell Ranger v5 and 10X Genomics pre-built mouse reference genome, which was modified to include the GFP sequence. Cell Ranger’s filtered feature by barcode matrices were then imported into R for downstream analysis. Low quality cells were flagged and excluded using the scuttle Bioconductor package (v1.0.0) with default parameters based on percentage of mitochondrial reads, library size, and number of genes detected per cell. Potential doublets were also excluded using the scDblFinder package from Bioconductor (v1.3.25). Cells that passed the quality control were imported into the Seurat R package (v4.0.2) for further analysis. We performed data normalization and feature selection using the SCTransform functionality with default settings from Seurat. Dimensionality reduction and data integration were subsequently performed using Pearson residuals obtained from SCTransform. Following unsupervised clustering of integrated data using FindNeighbors and FindClusters functionalities with default settings, we used literature-derived marker genes and cell type-specific signatures obtained from PanglaoDB database to annotate major cell types (24). We used the presto R package (https://github.com/immunogenomics/presto) that provides a fast implementation of Wilcoxon rank sum test and auROC analysis to further derive cluster- and cell type-specific marker genes, as well as to perform differential expression analysis between βA and Ctrl samples. Given that cells from the same sample are not independent observations, p-values obtained from Wilcoxon rank sum test can be too optimistic; therefore we used predictive power defined as abs(AUC-0.5)*2, along with log fold change for gene ranking. We defined the predictive power multiplied by logFC as the ranking statistic to perform pre-ranked GSEA of Hallmark pathways from MSigDB, using the fGSEA package (v1.15.2) from Bioconductor (25). For downregulated genes, the predictive power values were multiplied with -1 to indicate that the fold change in gene expression was negative. To further explore T cell states, we projected our data unto a published reference atlas of mouse T cell states (26), using the functionalities provided by the authors (https://github.com/carmonalab/ProjecTILs).

To assess gene ontologies enriched in *INHBA*-expressing human melanomas, we mined public data of the PanCancer Atlas Studies (https://www.cbioportal.org/). The mRNA Expression, RSEM (Batch normalized from Illumina HiSeq_RNASeqV2) patient data were separated into quartiles based on *INHBA* mRNA expression z-scores relative to all samples (log RNA Seq V2 RSEM). Only patients with the lowest (1^st^ quartile) and highest (4^th^ quartile) expression levels were compared. Genes significantly up-regulated (p<0.05 and logFC>0) in the highest *INHBA*-expressing tumors were used for gene ontology enrichment analysis in Enrichr (27).

### Intercellular communication analysis

In order to study intercellular communication, we used the nichenetr R package (v1.1.0). The analysis was aimed at predicting which target genes in receiver cell populations were most likely to be affected by changes in the expression of Inhba in tumor cells (sender cells). As a prior model of ligand-receptor, receptor-target and ligand-target interactions, we used the prebuilt ligand-target matrix (“https://zenodo.org/record/3260758/files/ligand_target_matrix.rds”), ligand-receptor network (“https://zenodo.org/record/3260758/files/lr_network.rds”) and weighted integrated network (“https://zenodo.org/record/3260758/files/weighted_networks.rds”). Gene symbols were converted from human to mouse based on one-to-one orthology. We considered receptors as active if expressed in receiver cells. As potential targets, we only considered genes expressed in at least 10% of cells in receiver populations, and which were also significantly differentially expressed (P-value ≤ 0.05, average fold change ≥ 0.5). Potential targets were ranked according to regulatory potential (from the prior model), and the top 100 were selected for representation in the circos plot (28).

### Bulk RNA barcoding and sequencing

For BRB-seq, YUMM3.3 cells were seeded in 6-well plates in quadruplicates at a density of 0.5x10^6^ per well and treated with 20 ng/ml IFN-γ and 40 ng/ml Activin-A individually or together for 4 or 12 hrs. Transcriptomes were determined by paired-end sequencing of a MERCURIUS BRB-seq library (Alithea Genomics) using an Illumina NovaSeq 6000 instrument. DEG analysis was performed using DESeq2 v1.36.0 (29). Raw read count matrices were normalized with the median of ratios normalization method implemented in DESeq2 that accounts for sequencing depth and RNA composition. Wald test was used for significance testing. For DEG analysis, adjusted p-values with a threshold of <0.05 were retained. For visualization or clustering, transformed versions of the count data were used using the Variance Stabilizing Transformations (VST) function. This function uses a statistical model to transform the raw count data (normalized by division by dimension or normalization factors), resulting in a matrix of values that have an approximately constant variance across the range of mean values. This normalization produces data transformed on a log2 scale and eliminates the dependence of the variance on the mean. Gene set enrichment analysis (GSEA) was performed using the GSEA function of the Bioconductor ClusterProfiler package (v4.4.4) and the following annotated gene set from MSigDB v6.2: the Hallmark gene set. Normalized enrichment scores and adjusted p-values were calculated separately for each comparison with the genes ranked according to sign(log2FoldChange)*-log10(pval) (25, 30).

The BRB-seq and scRNA-sec data in this publication have been deposited in NCBI’s Gene Expression Omnibus (31) and are accessible through GEO Series accession numbers GSE247229 (https://www.ncbi.nlm.nih.gov/geo/query/acc.cgi?acc=GSE247229) and GSE247228 (https://www.ncbi.nlm.nih.gov/geo/query/acc.cgi?acc=GSE247228, respectively.

### Statistical analysis

Statistical tests were performed using the Prism software (GraphPad). Unless indicated, data represent mean ± SEM of at least 2 independent experiments. When comparing two groups, normal distributions were analyzed by the Shapiro-Wilk normality test, and p-values calculated by Student’s t-test (normal distribution) or Mann-Whitney’s test (non-parametric test). One-way ANOVA was used to compare several groups of unpaired values. Data points identified as outliers by the regression and outlier (ROUT) removal method in Prism 9 with a False Discovery Rate ≤1% were excluded. Power Analysis was waved by the animal experimentation authorities due to pre-existing data about the effect sizes of Activin-A induced tumor growth and cachexia. Tumor volumes at the endpoint were compared by ANOVA or Student’s t-test, as indicated in the figure legends. Statistical significance of the effect of STING knockdown was independently validated using the Levene test to confirm the assumption that all groups have the same variance, and that the null hypothesis (same variance in all groups) was not rejected. Two-way ANOVA, analysis of the normality of its residuals, and computation of the Tukey Honest Significant Differences (Tukey HSD) to find the means that are significantly different from each other confirmed that there are only differences when comparing any group with the βA groups expressing shLuc.

## Results

### Activin-A induced changes in the cellular landscape of melanoma TME

**Figure 1:**
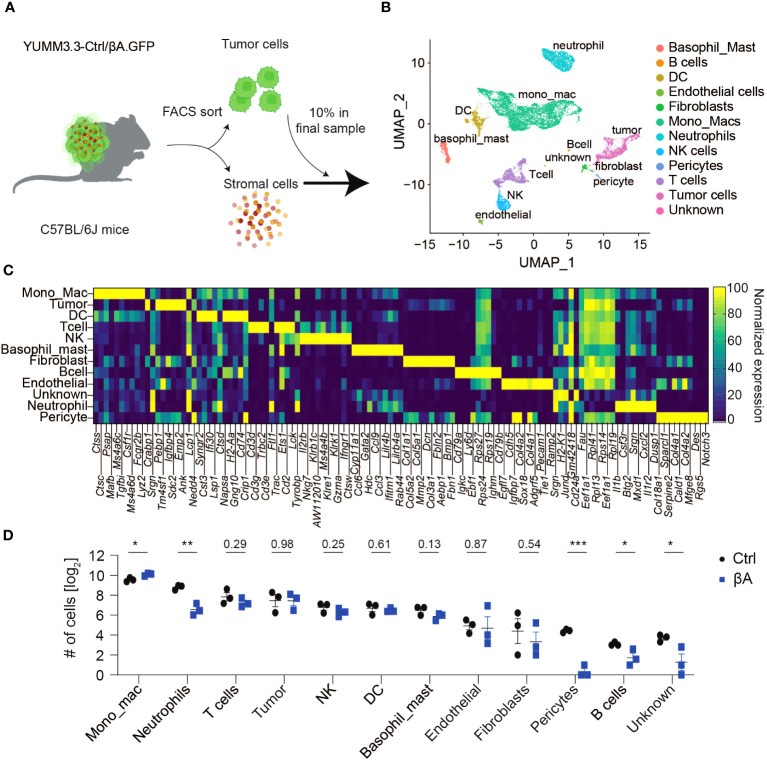
Characterization of YUMM3.3.GFP melanoma for scRNA-seq analysis. **(A)** Strategy to assess βA-induced changes in tumors by scRNA-seq: YUMM3.3-Ctrl and -βA tumors were dissociated into a single-cell suspension, and GFP^+^ tumor cells were sorted out by flow cytometry. Tumor cells were added back to account for 10% of cells in a final sample before analysis by 10x Genomics sequencing. **(B)** Uniform Manifold Approximation and Projection (UMAP) representation of annotated cell types identified in YUMM3.3 tumors. **(C)** Normalized expression of the top 10 genes used to identify different cell types in tumors. **(D)** Comparison of numbers of non-immune cells and unknown population (left panel), and immune cells (right panel) identified in Ctrl and βA tumors. Error bars, SEM (n = 3). *p<0.05, **p<0.01, ***p<0.001, Student’s t-test.

To assess activin-induced changes in melanoma and in their TME, YUMM3.3 mouse melanoma grafts expressing lentiviral *INHBA* (βA) or empty control lentivirus (Ctrl) were analyzed by scRNA-seq using the 10x Genomics platform (**Figure 1A**). To distinguish stromal and cancerous cells, the YUMM3.3-Ctrl and -βA melanoma cells were transduced with lentiviral green fluorescent protein (GFP) expression vector. Flow cytometry and Alamar blue assays confirmed that YUMM3.3-Ctrl and -βA melanoma cell lines each expressed similar levels of GFP and proliferated at comparable rates *in vitro* (**Supplementary Figures 1A, B**). Nevertheless, in syngeneic mice, tumor grafts of GFP-expressing YUMM3.3-βA tumors grew faster than Ctrl (**Supplementary Figure 1C**), as described previously for analogous grafts without GFP (17). To limit the variability among tumor sizes and its potential effect on gene signatures, triplicate Ctrl and βA samples consisting each of two tumors were collected on day 13 post-injection when tumor sizes were not yet significantly enlarged by Activin-A (**Supplementary Figures 1D, E**). To enrich the sequencing libraries for stromal cell transcripts, we separated single-cell suspensions into GFP^hi^ tumor cells and GFP^int/low^ stromal cells (**Supplementary Figure 1F**) and then mixed them at a 1:9 ratio. 10X Genomics sequencing yielded 155.8 and 153.5 x 10^6^ reads from a total of 2258 and 2017 cells on average per Ctrl and βA sample, respectively. Library sizes were around 67.8 and 77.1 x 10^3^ reads, and 2282 and 2894 genes per cell in Ctrl and βA samples, respectively (**Supplementary Figures 1G, H**, **Supplementary Table 1**). After filtering out low-quality cells and potential doublets, 25 distinct clusters corresponding to different cell types were identified by the unsupervised clustering of integrated data using FindNeighbors and FindClusters functionalities of Seurat R package (v4.0.2) (**Figures 1B, C**, **Supplementary Figures 1I–K**). Analysis of cell numbers revealed that monocytes and macrophages (MonoMacs) were most abundant (5500 cells) and significantly enriched in βA compared to Ctrl tumors (**Figure 1D**). On the other hand, neutrophils which were the second-most abundant population (1644 cells), as well as rare B cells (37 cells) were instead enriched in Ctrl samples. Other relatively rare cell types included 158 fibroblasts, 134 endothelial cells, and 69 pericytes. Of note, while βA did not affect the number of fibroblasts or endothelial cells, it almost completely depleted pericytes, as well as a distinct cluster of only 50 monocytic progenitor-like cells that we named unknown because they lacked definitive MonoMacs markers (**Figures 1D**, **Supplementary Figures 1L–N**). By contrast, the number of tumor-infiltrating T cells, dendritic cells (DCs), natural killer (NK) cells, or basophils (1185, 1113, 600, 585, or 460 cells, respectively) were not significantly altered by βA compared to Ctrl at the early stage examined.

### Intercellular communication analysis reveals Activin-responsive cells in the TME

**Figure 2:**
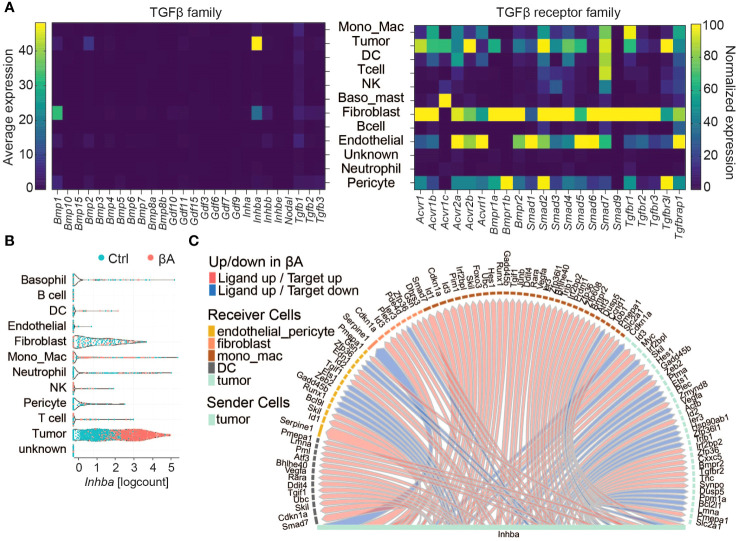
Characterization of Activin-A responding cells in the TME. **(A)** Left: Heatmap displaying the average expression of TGF-β family members (columns) in different cell types (rows). Right: Normalized expression of TGF-β family receptors (columns) in cell types (rows). **(B)** Violin plot of INHBA expression in cell types of Ctrl and βA expressing YUMM3.3.GFP tumors. **(C)** Intercellular communication analysis of Activin-A responsive cells in the TME.

To explore which cell types may respond to Activin-A and/or to other TGF-β family members, we first assessed the expression of TGF-β-related ligands and their receptors. Analysis across cell types revealed the highest expression of *Inhba* in tumor cells, as expected due to lentiviral overexpression (**Figures 2A, B**). Additionally, tumor cells expressed *Bmp2* and low levels of *Gdf11*, *Bmp4*, and *Tgfb1* (**Figures 2A, B**; **Supplementary Table 2**). The most significant source of TGF-β related ligands were fibroblasts, which mostly expressed *Inhba*, followed by *Inhbb*, *Tgfb1/2/3*, *Gdf10* and *Gdf11* alongside the tolloid-like metalloprotease encoded by *Bmp1*. *Tgfb1* was also transcribed at low levels in all other cell types, except in B cells, and in neutrophils. Fibroblasts also expressed the highest levels of type I and II activin, TGF-β and BMP receptors, except *Acvr2b* and *Acvr1c* mRNAs. *Acvr2b* was even more highly transcribed in tumor cells. *Acvr1c* mRNA was most abundant in basophils, followed by tumor and NK cells, but below detection in fibroblasts. *Acvr1b* encoding the type I activin receptor ALK4 was highly expressed in the clusters of fibroblasts, tumor cells, MonoMacs, DCs, endothelial cells, and pericytes. Similar expression patterns were observed for *Acvr2a/b* receptors and *Smad2/3/4*, except for DCs that show lower levels of *Acvr2b* and *Smad3* transcripts (**Figures 2A, B**; **Supplementary Table 2**). To address which cells in the TME respond to Activin-A, we investigated active ligand-target interactions between cells using the computational method NicheNetR (28). The analysis revealed activin-induced signaling in tumor cells, DCs, MonoMacs, fibroblasts, and endothelial and pericyte populations (**Figure 2C**). Gene ontology enrichment analysis in tumor cells revealed upregulation of developmental programs such as bone, heart, neuron, and endoderm formation, pseudo-epithelial-mesenchymal transition (EMT) and ECM organization, alongside increased IFN responses, antigen processing, and MHC I presentation (**Supplementary Figures 2A, B**). Upregulation of a pseudo-EMT signature and of known Smad2,3 target genes such as *Pmepa1* and *Skil* within the tumor cells (**Figure 2C**) is consistent with increased autocrine Activin-A signaling.

### Activin-A secretion by melanoma cells alters several cancer hallmark signatures across the TME

**Figure 3:**
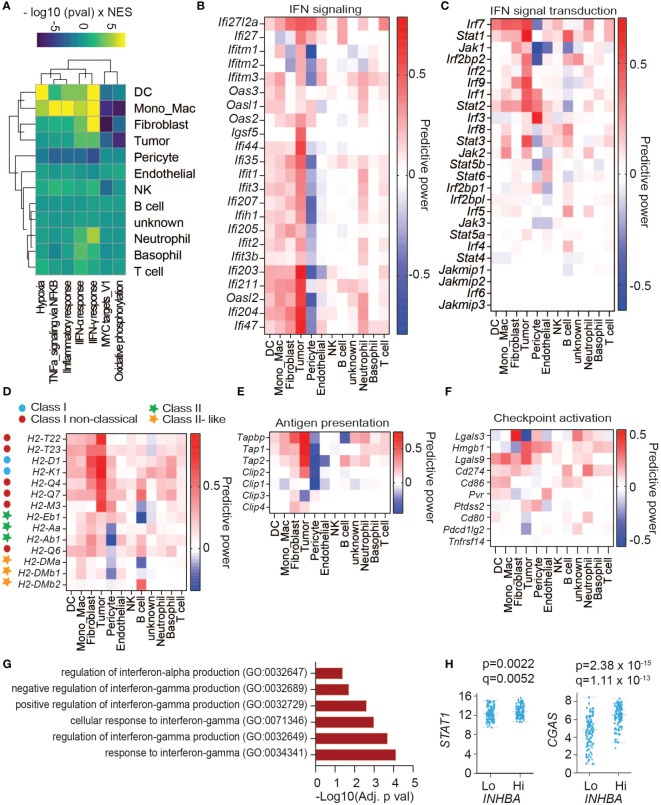
Activin-associated increase in IFN signature does not lead to higher CD8^+^ T cell infiltration. **(A)** Heatmap showing top changes in enrichment of cancer Hallmark gene sets (rows) in cell types (columns) induced by melanoma-derived Activin-A. **(B)** Heatmap showing the predictive power of differentially regulated genes of IFN signaling hallmark signatures. Predictive power was defined as 2 * sign(logFC) * abs(AUC- 0.5), reflecting the directionality of change as well as the discriminatory power. **(C–F)** Heatmaps showing the predictive power of differentially regulated **(C)** genes of IFN signaling pathway, **(D)** MHC genes, **(E)** genes encoding enzymes that mediate antigen processing, and **(F)** genes of ligands for immune checkpoints activation. **(G, H)** GO biological processes enriched among up-regulated genes in PanCancer melanoma sample expressing the highest levels of *INHBA* **(G)**, and **(H)** differential expression of *STAT1* and *CGAS* in high compared to low *INHBA*-expressing melanoma tumors.

To survey activin-induced changes in the TME related to cancer, we performed gene set enrichment analyses using curated Hallmark gene sets (32). As shown in **Figure 3A**, the cell types with the most significant changes in these Hallmark signatures in βA compared to Ctrl tumors were DCs, followed by MonoMacs, fibroblasts, tumor cells, and pericytes, whereas T cells were changed the least, consistent with predicted target cells from the intercellular communication analysis. In βA tumors, DCs and MonoMacs, together with almost all other cell types, also significantly increased the signature of hypoxia (**Figure 3A**, **Supplementary Figure 3A**). A notable exception were pericytes and tumor cells, where several hypoxia response genes were instead downregulated. On the other hand, the signatures Oxidative phosphorylation and Myc Targets were significantly decreased by βA, especially in fibroblasts, MonoMacs, DCs, and within the tumor cells themselves (**Figure 3A**, **Supplementary Figures 3B, C**). Remarkably, aside from pericytes, all cell types in βA tumors showed an elevated inflammatory response signature and signs of increased type I and type II interferon signaling, especially DCs, followed by MonoMacs, fibroblasts, and tumor cells (**Figures 3A, B**). Concomitantly, MonoMacs, fibroblasts, and tumor cells showed increased TNFA signaling via NFκB in βA as compared to Ctrl tumors (**Figure 3A**, **Supplementary Figure 3D**).

### Activin-A upregulates IFN signaling in melanoma

Interferon signal transduction is mediated by JAK, STAT, and IRF proteins (33). Analysis of differentially expressed genes (DEGs) showed upregulation of several IFN signal transduction components across cell types, including *Irf7*, *Stat1*, *Stat2*, and to a lesser extent *Irf1/2/9* and *Jak1/2* expression (**Figure 3C**). Among these, upregulation of *Stat1* expression was most significant within the tumor cells themselves. Key components of an IFN signature involve antigen presentation and immune checkpoints. Analysis of genes involved in antigen processing and presentation showed that βA-expressing tumors upregulated the expression of classical and non-classical MHC-I genes across different cell types, especially in the *INHBA*-expressing tumor cells themselves (**Figure 3D**). *INHBA*-expressing Tumor cells also increased their expression of Transporter Associated With Antigen Processing (TAP) genes responsible for MHC I antigen loading (**Figure 3E**). A survey of immune checkpoint receptor ligands revealed upregulation of *Lgals3* in fibroblasts, *Hmgb1* in tumor cells, and *Lgals9* and *Cd274* across cell clusters (**Figure 3F**). These data point to increased activation of the corresponding immune checkpoint receptors LAG3, PD1, and TIM3 in βA-expressing tumors. To address whether *INHBA* expression correlates with increased IFN signaling also in human melanoma, we analyzed the Melanoma PanCancer database. A comparison of tumors expressing low or high *INHBA* mRNA levels, combined with gene ontology analysis of upregulated genes revealed that *INHBA*-high tumors upregulate signatures related to cellular responses to type I and II IFNs in (**Figure 3G**), as well as *STAT1* and *CGAS* expression (**Figure 3H**). These results suggest that Activin-A enhances IFN signaling also in human tumors.

### *INHBA*-induced alterations in the expression of chemokine and cytokine mRNAs

As the most significantly upregulated Hallmark gene sets in βA tumors were related to inflammatory responses, we systematically surveyed changes in the expression of proinflammatory chemokines, cytokines, and matrix metalloproteases (MMPs). Among cytokines, βA most significantly increased the expression of *Il1b, Il18 Il33*, and *Il15*, both within tumor cells and fibroblasts. *Il1b* expression also increased in DCs and MonoMacs, whereas βA-expressing tumor cells showed also elevated levels of *Il16* mRNA (**Supplementary Figures 4A, B**). Notably, *Ifng* expression was comparable between the groups (**Supplementary Figure 4B**). In keeping with the increased enrichment of the IFN signaling gene set described above (**Figure 3A**), multiple cell types in βA tumors also upregulated the IFN-γ inducible *Cxcl9*, *Cxcl10* and *Ccl5* genes.

Despite the observed increase in their mRNA levels, a previous analysis established that secretion of CXCL9 and CXCL10 proteins is not increased by Activin-A, but rather decreased both in tumors and in an activated mouse DC1 cell line (17) (**Supplementary Figure 4C**). CXCL9 and CXCL10 can be degraded by cysteine proteases of the cathepsin family (34). To address if Activin-A modulates chemokine degradation by cysteine cathepsins, we treated DC1 cells with the cysteine protease inhibitor E64 or with the cathepsin S inhibitor Petesicatib. Treatment of DC1 cells with Activin-A diminished CXCL9 protein levels in DC1 cells regardless of the presence of any of these inhibitors, suggesting that cysteine proteases or cathepsin S are unlikely responsible (**Supplementary Figures 4D–F**).

Cytokine and chemokine activities may be regulated directly or indirectly through the remodeling of ECM by MMPs (35). Among MMPs, we observed that βA-expressing tumors significantly upregulated MMP2, 3, 14, and 19 (**Supplementary Figure 4G**). However, potential interactions of these or other proteases with chemokines remain to be explored.

### Despite elevating IFN signaling, Activin-A does not promote T cell responses

**Figure 4:**
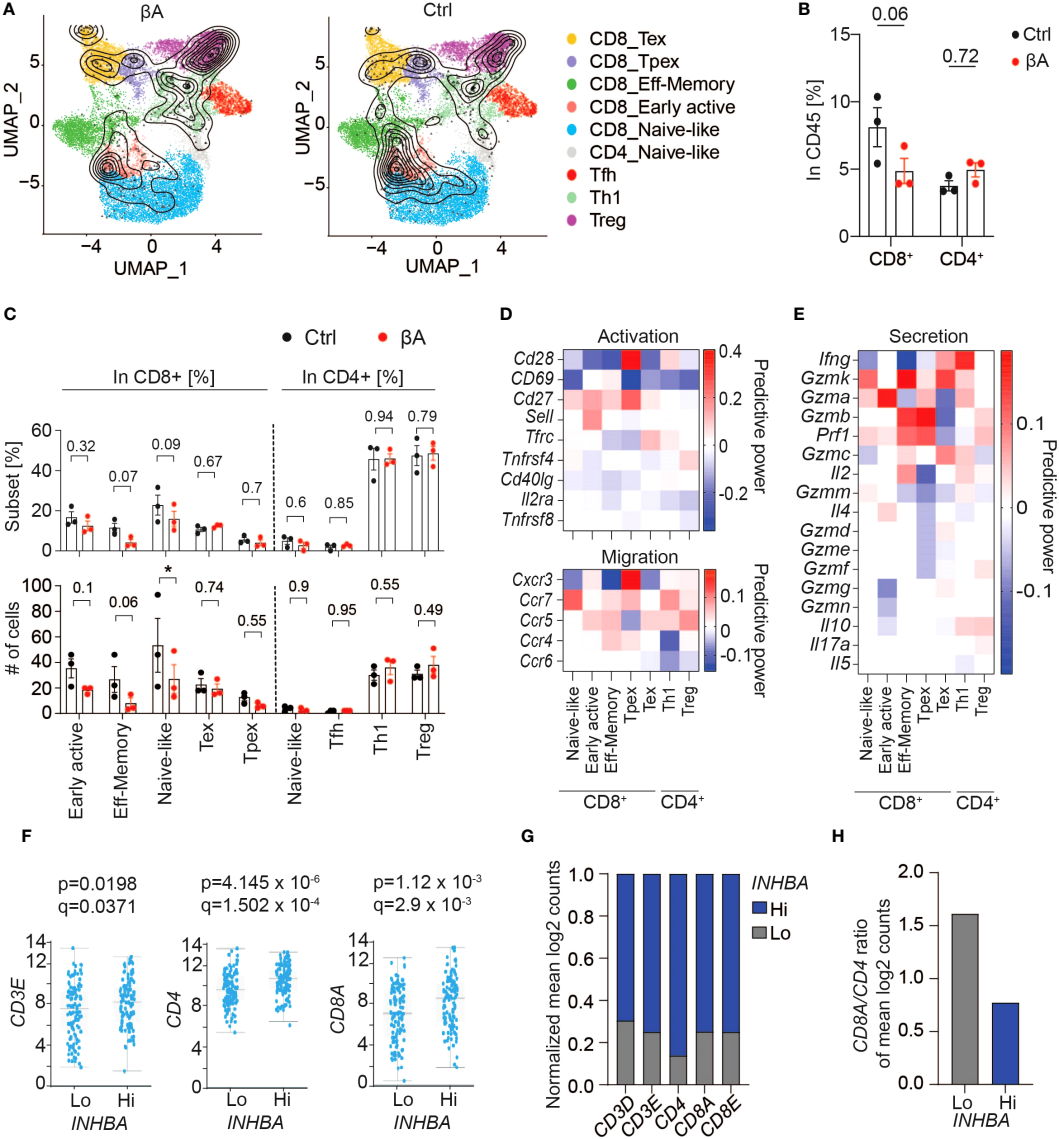
Activin-A expression in tumors does not lead to increased CD8 T cell activation. **(A)** UMAP representing βA (left) or Ctrl (right) T cell clusters projected onto a reference mouse T cell atlas (26). **(B)** Numbers of CD8^+^ or CD4^+^ T cells in βA compared to Ctrl conditions (n = 3). **(C)** Frequencies (top) and numbers (bottom) of T cell subtypes from **(A)** in βA compared to Ctrl conditions. Error bars, SEM (n = 4-5); *p<0.05. Student’s t-test. **(D, E)** Heatmaps showing changes in expression of [**(D)**, top panel] activation markers, [**(D)**, bottom panel] chemokine receptors, and **(E)** secretion profile in T cell subtypes in βA compared to Ctrl conditions. **(F)** TCGA database analysis of differential expression of *CD3E, CD4*, and *CD8A* in high compared to low *INHBA*-expressing melanomas (scale, mean log2 counts). **(G, H)** Mean log2 counts of *CD3D*, *CD3E*, *CD4*, *CD8A*, and *CD8E* transcripts normalized to total number of trancripts in both groups **(G)**, and **(H)** ratios of *CD8A* to *CD4* log2 counts in Hi *versus* Lo *INHBA*-expressing melanomas in TCGA database.

To survey the composition of tumor-infiltrating T cell subsets, we projected our scRNA-seq data onto a reference atlas of T cell signatures (26). Already at the early stage of tumor growth examined here by scRNA-seq, we observed a strong trend for reduced CD8^+^ T cell infiltration in βA compared to Ctrl tumors, whereas the number of CD4^+^ T cells was unchanged (**Figures 4A, B**). Quantification of T cell subtypes based on their gene signatures showed that βA expression reduced the intratumoral accumulation of naïve-like T cells, whereas a trend for a decrease in effector memory T cells and early active CD8^+^ T cells did not reach statistical significance at this stage, and the number of CD4^+^ Tregs was unchanged compared to Ctrl (**Figure 4C**). Interestingly, T cells in βA tumors showed reduced expression of the activation markers CD28 and/or CD69, and analysis of known secreted effector molecules and of selected markers of cell migration revealed that their power values all remained below 0.2, suggesting that they were only minimally changed, or not at all (**Figures 4D, E**). Observed changes in the gene expression of NK cells were also minimal at the stage examined, with only 25 genes showing power values higher then 0.2 (**Supplementary Figure 4H**).

To investigate a possible influence of Activin-A on CD8^+^ T cell infiltration in melanoma patients, we analyzed the expression of CD3, CD4, and CD8 as a proxy of T cell infiltration in the PanCancer Melanoma database. Despite an overall increase in T cell infiltration (**Figures 4F, G**), human melanoma with high levels of *INHBA* had a two-fold lower CD8 to CD4 ratio (**Figure 4H**). Taken together, these data support the notion that Activin-A in melanoma diminishes intratumoral CD8^+^ compared to CD4^+^ T cells, and despite increased IFN signaling across the TME.

### Autocrine Activin-A signaling counteracts cytostatic IFN-γ activity in YUMM3.3 cells while augmenting STAT1 activation

**Figure 5:**
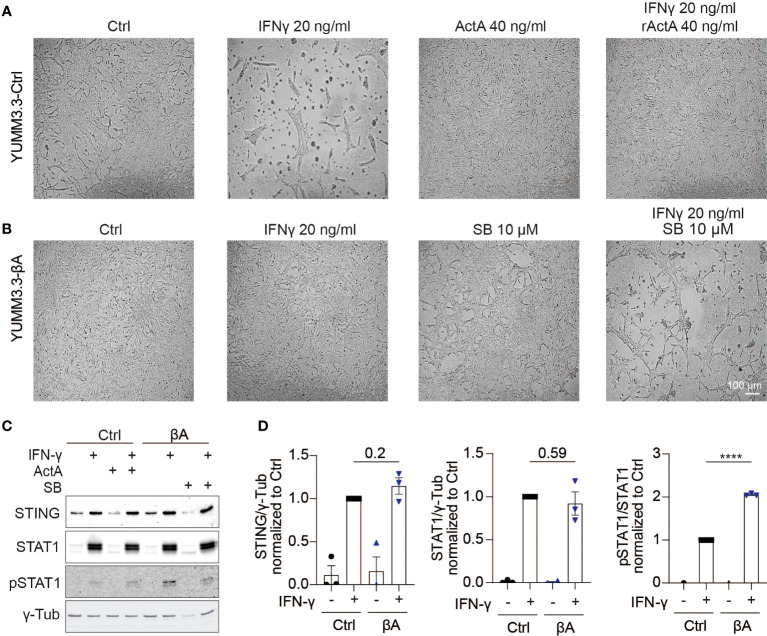
Activin-A promotes IFNγ-induced Stat1 activation in YUMM3.3 cells. **(A, B)** Representative images of **(A)** YUMM3.3-Ctrl or **(B)** YUMM3.3-βA cells treated for 48 hrs with 40 ng/ml Activin-A, 20 ng/ml IFN-γ or 10 μM SB-431542 (SB) where indicated (scale bar, 100 µm). **(C)** Representative Western blots of STING, STAT1, pSTAT1, and γ-Tubulin (loading control) in YUMM3.3-Ctrl and -βA cells treated during 24 h with 20 ng/ml IFN-γ, 40 ng/ml Activin-A or 10 μM SB-431542 where indicated. **(D)** Quantification of STING, STAT1, and pSTAT1 levels Western blots relative to γ-Tubulin in YUMM3.3-Ctrl and -βA treated with 20 ng/ml IFN-γ or control (H_2_O) during 24 h. Error bars, SEM (n = 3); ****p<0.0001, Student’s t-test.

Since Activin-A secretion by melanoma grafts stimulated an IFN signature not only in the TME but also within the tumor cells themselves, we investigated whether Activin-A could directly modify the impact of IFN-γ on cultured melanoma cells. To address this, we treated YUMM3.3-Ctrl cells for 48 hrs with IFN-γ alone or together with recombinant Activin-A. In the presence of IFN-γ alone, YUMM3.3-Ctrl cells congregated in abnormal clumps and grew approximately 1.7-fold less than if treated with vehicle control, whereas co-treatment with Activin-A suppressed these effects (**Figure 5A**, **Supplementary Figures 5A, B**). Co-treatment with Activin-A conferred similar resistance to cytostatic IFN-γ activity also in parental YUMM3.3 cells, ruling out a possible artifact linked to lentiviral transduction (**Supplementary Figures 5A, B**). In good agreement, cytostatic activity of IFN-γ was also abolished by lentiviral *INHBA* expression in YUMM3.3-βA cells, whereas co-treatment with the ALK4 inhibitor SB-431542 rescued it (**Figure 5B**). To test if autocrine Activin-A signaling in YUMM3.3-βA cells interferes with IFN-γ signal transduction, we monitored the IFN-γ induced activation of STAT1 and the expression of STING. Western blot analysis revealed that IFN-γ treatment for 24 hrs similarly increased STAT1 and STING expression in both YUMM3.3-Ctrl and -βA cells (**Figures 5C, D**). By contrast, the levels of STAT1 phosphorylation induced by IFN-γ increased 2-fold in βA compared to Ctrl cells.

To compare the contributions of STAT1 and other candidate effectors to IFN-γ induced growth inhibition, we treated cells with the STAT1/3/5 inhibitors Fludarabine and Nifuroxazide, or with the JAK1/2 inhibitor Ruxolitinib. Co-administration with either Fludarabine or Nifuroxazide enhanced the growth inhibitory effect of IFN-γ, rather than suppressing it (**Supplementary Figure 5B**). In sharp contrast, incubation of IFN-γ treated cells with Ruxolitinib restored their proliferation as efficiently as co-treatment with Activin-A. To address if Activin-A similarly increases the resistance of cancer cells to chemotherapy, we treated YUMM3.3 cells with Carboplatin together with or without Activin-A. Analysis of the proliferation of viable cells by Alamar Blue staining showed that carboplatin treatment inhibited cell proliferation regardless of the presence of Activin-A (**Supplementary Figure 5C**). Collectively, these results indicate that crosstalk with Activin-A signaling can augment STAT1 activation by IFN-γ and confer resistance to anti-proliferative IFN-γ/JAK signaling within melanoma cells.

### Activin-A dampens the IFN-γ response in melanoma cells *in vitro* instead of stimulating it

**Figure 6:**
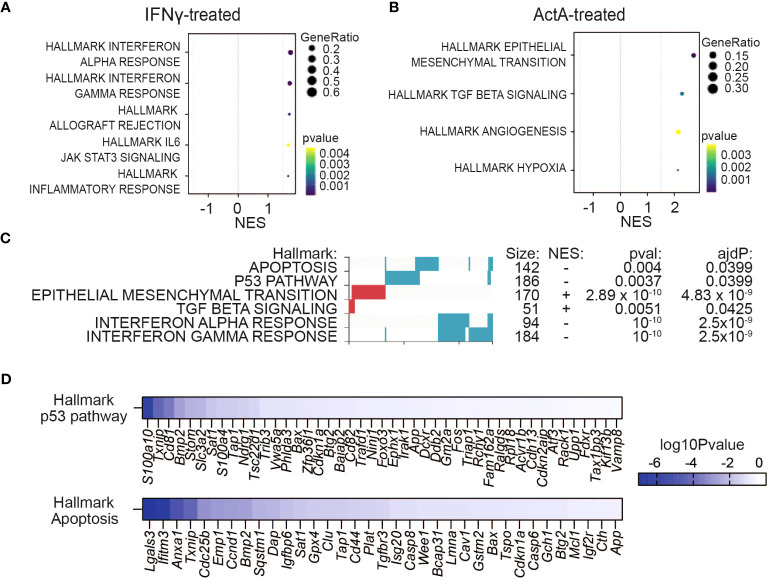
Activin-A precludes IFNγ-induced cytostatic effect in melanoma cells without directly augmenting IFN gene signatures *in vitro*. **(A, B)** GSEA plots showing enriched hallmark pathways in YUMM3.3 cells treated with 50 ng/ml ActA **(A)** or 20 ng/ml IFN-γ **(B)** during 12 hrs. **(C)** GSEA analysis showing differential regulation of Hallmark gene set signatures (red, upregulated; blue downregulated) in YUMM3.3 cells treated 12 hrs with 20 ng/ml IFN-γ plus 50 ng/ml ActA, versus IFN-γ alone. **(D)** Genes leading the p53 and apoptosis hallmarks downregulation by the Activin-A/IFN-γ combo compared to treatment with IFN-γ alone.

To assess how crosstalk of Activin-A with IFN-γ influences gene expression in the absence of the TME, mRNA from YUMM3.3 cells that were treated with IFN-γ and Activin-A individually or together for 4 or 12 hrs was analyzed by Bulk RNA Barcoding and Sequencing (BRB-seq) (36). Analysis of DEGs (adjusted p-val <0.05) revealed that IFN-γ treatment alone altered the expression of 551 genes, compared to 123 activin-regulated genes, and that co-administration of both factors changed only 53 genes compared to IFN-γ alone (**Supplementary Figures 6A–C**). Furthermore, gene set enrichment analysis (GSEA) of these DEGs showed that the hallmark signatures IFN Responses, Allograft Rejection, JAK/STAT3 signaling, and Inflammatory Response were specifically upregulated by IFN-γ (**Figure 6A**), whereas treatment with Activin-A alone instead stimulated the cancer hallmark signatures of EMT, angiogenesis, and hypoxia, alongside a “TGF-β signaling” signature genes (**Figure 6B**). Activin-A also induced the EMT and TGF-β signatures when administered together with IFN-γ, which is expected given that Activin-A and TGF-β activate the same Smad transcription factors. However, compared to cells treated with IFN-γ alone, the cells receiving IFN-γ together with Activin-A differentially enriched their interferon-gamma and alpha responses, as well as apoptosis and p53 pathway signatures (**Figures 6C, D**, **Supplementary Figure 6D**). To assess if Activin-A antagonizes the cytostatic activity of IFN-γ by interfering with cell death, we conducted Alamar Blue assays on cells that were co-treated with the pan-caspase inhibitor Z-VAD-FMK, or with the necroptosis or ferroptosis inhibitors Nec1s or Ferrostatin-1, respectively, together with IFN-γ and with or without Activin-A. We found that IFN-γ treatment severely diminished YUMM3.3 cell proliferation regardless of the presence of these cell death inhibitors, and that the presence of Activin-A largely restores cell proliferation (**Supplementary Figures 6E, F**). Conversely, the addition of Activin-A could not rescue the survival and proliferation of YUMM3.3 cells that were treated with the ferroptosis-inducing agent Erastin (**Supplementary Figure 6F**). These data suggest that Activin-A protects YUMM3.3 cells against inhibition of cell proliferation by IFN-γ likely via a mechanism other than by inhibiting apoptosis, necroptosis or ferroptosis.

To test if IFN-γ interferes with the cell cycle, we transduced YUMM3.3 cells with the fluorescence ubiquitination cell cycle indicator (FUCCI) (37) and assessed the percentages of cells in G1 or G2/S/M phase upon treatment with IFN-γ that were co-treated or not with Activin-A. We found that in YUMM3.3 cells that survived in the presence of IFN-γ, Activin-A did not change the cell cycle progression (**Supplementary Figure 6G**). To address how many of these genes might be directly deregulated by Activin-A in tumor cells also *in vivo*, we compared the DEGs that are common in YUMM3.3 cells both in culture and in tumor grafts. We observed that only 1.25% of DEGs in engrafted tumor cells overlapped with the 123 DEGs that were also regulated by Activin-A treatment in cultured cells (**Supplementary Figures 6H, I**). Importantly, this overlap did not include IFN-regulated genes (**Supplementary Table 3**). These data suggest that stimulation of the IFN signature by Activin-A *in vivo* likely depends primarily on the TME.

### Activin-A tumor-promoting role is dependent on STING in YUMM3.3 cells

**Figure 7:**
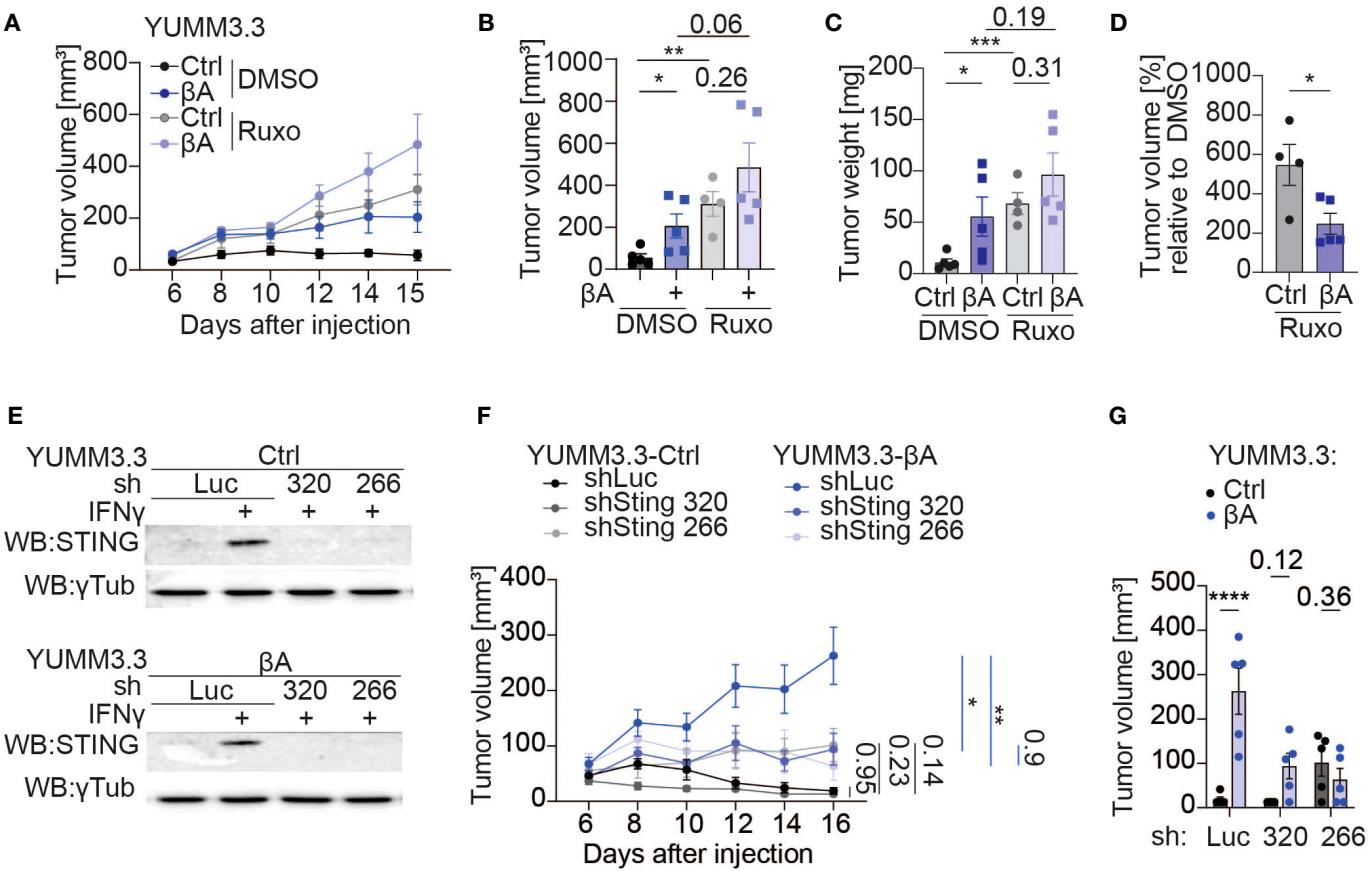
Sting knockdown partially diminishes the tumor-promoting function of Activin-A. **(A–D)** Growth curves **(A)**, individual tumor volumes **(B)**, tumor weights **(C)**, and tumor volumes of treatment groups normalized to a vehicle **(D)** at the endpoint of YUMM3.3-Ctrl and YUMM3.3-βA tumor grafts in C57BL/6J mice after treatment with 1 mg of Ruxolitinib, or DMSO control. Error bars, SEM (n=5 per genotype); **p<0.01, ***p<0.001, ordinary one-way ANOVA with Holm-Šídák correction for multiple comparisons. **(E)** Western blot analysis of STING protein levels in YUMM3.3-Ctrl (top) and YUMM3.3-βA cells (bottom) that were stimulated with 20 ng/ml IFN-γ for 12 hrs after stable transduction with the indicated lentiviral shRNA expression vectors. Analysis of γ-Tubulin served as a loading control. **(F, G)** Growth curves **(F)** and tumor volumes at the endpoint **(G)** of syngeneic YUMM3.3-Ctrl and YUMM3.3-βA melanoma grafts expressing shLuc, shSting 320 or shSting 266. Error bars, SEM (n=5 per genotype); *p<0.05, **p<0.01, ****p<0.0001, two-way ANOVA with Holm-Šídák correction for multiple comparisons.

To assess the contribution of IFN signaling to Activin-A induced tumor growth, mice bearing YUMM3.3-Ctrl or YUMM3.3-βA tumors were treated with the JAK1/2 inhibitor Ruxolitinib or with empty vehicle control (DMSO). In the DMSO-treated group, βA-expressing tumors retained their expected growth advantage compared to Ctrl. By contrast, in Ruxolitinib-treated hosts, Ctrl tumors grew almost as fast as βA tumors (**Figures 7A, B**). Thus, in presence of Ruxolitinib, a trend for βA to still accelerate both the volume and the weight of tumors no longer reached statistical significance (**Figures 7B, C**). To further demonstrate the significant reduction in JAK-mediated tumor immune surveillance by βA, we also plotted the volumes of Ruxolitinib-treated tumors at the endpoint relative to the volumes of analogous tumors in DMSO-treated mice. We found that Ruxolitinib treatment increased the growth of Ctrl tumors by 546%, compared to only 247% in βA tumors, which corresponds to a 2.2-fold difference (**Figure 7D**). These results indicate that Activin-A promotes tumor growth at least in part by interfering with anti-tumoral JAK1/2.

JAK1/2 signaling is necessary to mount effective anti-tumor responses, which in turn are required to slow the growth of Ctrl compared to βA-expressing melanoma (17, 38). Considering that Activin-A secretion in YUMM3.3-βA tumors did not increase *Ifng* transcription at the stage examined, we hypothesized that its tumor-promoting function may involve STING activation, because STING can upregulate an IFN signature independently of IFN-γ via type I interferons (39). To address this, we transduced YUMM3.3-Ctrl and YUMM3.3-βA cells with shRNAs 320 or 266 targeting *Sting*, or with luciferase (shLuc) as a control. Upon treatment with IFN-γ, STING protein accumulated specifically in shLuc-transduced cells both in the presence and absence of the βA transgene, but not in sh320 or sh266 cells, confirming efficient KD (**Figure 7E**, **Supplementary Figure 7A**). Knock-down of *Sting* did not impair YUMM3.3 cell proliferation or suppress its inhibition by IFN-γ in Alamar Blue assays (**Supplementary Figure 7B**). To investigate the influence of STING in tumor cells on the effect of Activin-A *in vivo*, we grafted YUMM3.3-ctrl or βA cells expressing shSting 320 or 266 or shLuc in syngeneic mice. Analysis of tumor growth curves revealed that shLuc-expressing cells only efficiently formed tumors if they also express βA, confirming that immune protection and its possible stimulation by shRNA vector can be potently inhibited by Activin-A (40). Compared to shLuc, *Sting* shRNAs did not significantly influence the growth of YUMM3.3-Ctrl tumors. However, they specifically reduced the growth of βA tumors. Consequently, Ctrl and βA tumors depleted of STING grew at comparable rates (**Figures 7F, G**). These results suggest that the tumor-promoting role of Activin-A depends on pernicious STING activation in tumor cells.

## Discussion

Previously, we reported that Activin-A secretion by melanoma cells promoted tumor growth in three independent syngeneic grafting models by facilitating CD8^+^ T cell exclusion (16, 17). Here, transcriptional profiling by scRNA-seq in one of these models at a stage prior to overt CD8 T cell exclusion revealed that Activin-A first enriched a population of monocytes and macrophages (MonoMacs) at the expense of neutrophils, coinciding with prominent transcriptional changes within the cancer cells themselves, DC, MonoMacs, and fibroblast populations. Interestingly, among several significantly altered hallmark gene set signatures of cancer, Activin-A secreting tumors most prominently upregulated IFN pathways across multiple cell types. Further emphasizing an intersection with IFN signaling, Activin-A also modulated an IFN-γ response within these melanoma cells *in vitro*, as shown by increased STAT1 phosphorylation accompanied by a marked attenuation of IFN-γ induced cytostasis. Finally, knockdown of *Sting* and, to a lesser extent, pharmacological inhibition of JAK diminished the tumor growth-promoting effect of Activin-A in syngeneic grafts. Besides a first snapshot of Activin-A induced changes in the TME of a solid cancer at single-cell resolution, these findings provide proof of principle that the associated immune evasion and tumor growth can be mitigated by targeting a novel interaction with the STING pathway.

Depending on the context, Activin-A signaling can be pro- or anti-inflammatory in multiple cell types of the innate and adaptive immune systems during infectious and autoimmune diseases, and in allergic disorders (9). However, available insights into the roles of Activin-A in regulating anti-tumor immunity are scarce. Here, transcriptional profiling of the widely used *Braf* mutant syngeneic YUMM3.3 mouse melanoma model showed that gain of Activin-A secretion enriched MonoMacs population at the expense of neutrophil infiltration already at an early stage of tumor growth. An increase of monocyte and macrophage populations by cancer cell-derived Activin-A has also been observed by flow cytometry in YUMM3.3 and iBIP2 melanoma, and in skin cancer models (17, 19). Depletion of neutrophils by Activin-A to our knowledge has not been described in these or other cancer types, but is consistent with an inhibitory effect of Activin-A on the chemotaxis and recruitment of neutrophils to *S. aureus* infection sites in mouse skin (41). In our dataset, neutrophils expressed only very low levels of activin receptors, though, suggesting that Activin-A may deplete neutrophils indirectly. Indeed, ligand-target interaction analysis of Activin-A responsive cells in the TME identified no neutrophils, but DCs, MonoMacs, Fibroblasts, Pericytes, and Endothelial and Tumor cells, all of which also transcribed activin type I and II receptors. Thus, Activin-A may promote immune suppression and the associated tumor growth by several mechanisms, including remodeling of innate immune responses and of the perivascular niche, alongside changes in the transcriptome of tumor cells.

Interestingly, while Activin-A secretion by the melanoma cells did not significantly alter the numbers of fibroblasts or endothelial cells, it almost completely depleted the pericytes. Since pericytes are crucial for the maturation and function of microvessels, future studies should investigate how Activin-A diminished their numbers, and how this depletion might influence tumor vascularization. In endothelial cells, Activin-A can directly inhibit cell proliferation (42–44), or stimulate it by promoting VEGF-A expression (45, 46). Although we observed no upregulation of *Vgfa* in endothelial cells of YUMM3.3 tumors, *INHBA* overexpression also promotes tumor vascularization in B16-F1 melanoma and in transgenic mouse models of skin squamous cell carcinoma, where this effect was enhanced by a polarizing effect on tumor-associated macrophages (16, 19). Whether tumor vascularization is similarly enhanced by Activin-A in YUMM3.3 melanoma grafts, or whether a proangiogenic activity can be masked by the depletion of pericytes warrants further study. Here, we also did not further investigate a role in fibroblasts because these cells were scarce. However, fibroblast activation by Activin-A plays a crucial role in wound healing, scarring and extracellular matrix remodeling (47, 48). Furthermore, fibroblasts that are activated by *INHBA* expression in keratinocytes of human papilloma virus-induced skin tumors have been shown to promote tumor cell migration, angiogenesis and an inflammatory response gene signature (49). Activin-A signaling is also increased in cancer-associated fibroblasts of the invasive niche in human basal cell carcinoma (21). Consistent with these studies, we found that fibroblasts in *INHBA*-overexpressing melanoma increased their expression of chemokines, cytokines, inflammatory responses, and hallmark signatures associated with TNF signaling.

Gene set enrichment analysis showed the most pronounced alteration of cancer hallmark gene set signatures in DCs and MonoMacs. A previous *ex vivo* analysis established that monocyte-derived DC subsets can also secrete Activin-A by themselves to thereby promote their phagocytic activity (50) while dampening MHC-II expression and T cell activation (51) and the LPS-induced secretion of the chemokines CCL2, CXCL8, and CXCL10, and of IL-6 and IL-12p70 (52). Furthermore, analysis of DCs derived from the bone marrow of *Inha* ^-/-^ mice lacking the activin receptor antagonist inhibin indicated that one or several endogenous activins dampen DC maturation both *in vitro* and *in vivo* (53). Here, Activin-A secretion by YUMM3.3 cells did not diminish MHC I expression in DCs but rather increased it. Furthermore, MHC II gene expression was unchanged, concurring with the fact that a tolerogenic downregulation of MHC II in lymph node DCs by Activin-A in a mouse model of allergic airway disease is indirect and mediated by the induction of Tregs (54). In good agreement, melanoma cell-derived Activin-A also did not induce Tregs or impair the maturation of cDC1 cells (17).

Our previous cytokine and chemokine profiling in short term cultures of dissociated YUMM3.3 melanoma grafts showed that Activin-A secretion by the cancer cells diminishes both the levels of IFN-γ and the secretion of CXCL9 and CXCL10 in the conditioned medium (17). Quantification of intracellular CXCL10 staining by flow cytometry confirmed that Activin-A signaling also downregulated CXCL10 in intratumoral macrophages of *INHBA*-expressing tumors, and in murine cDC1 cells. However, as shown in the present study, *INHBA* overexpression in YUMM3.3 tumors did not result in a corresponding decrease in *Cxcl9* or *Cxcl10* mRNA levels, strongly suggesting that the expression of these chemokines is regulated post-transcriptionally. The functional relevance of Activin-A signaling in DCs for its tumor-promoting function in melanoma or other cancers, and possible mechanisms of how it attenuates CXCL9/10 secretion in myeloid lineages warrant further investigation. Separate studies will also be needed to define the precise roles of activin signaling in tumor-associated macrophage subsets. Antibody depletion of CSF1R^+^ macrophages in *INHBA*-driven skin squamous cell carcinoma delayed the onset of tumor growth and decreased tumor vascularization (19). By contrast, depletion of this subset in YUMM3.3 melanoma did not diminish the tumor-promoting activity of Activin-A (17). However, a possible tumor-promoting role for other MonoMacs populations cannot be excluded.

Within the melanoma cells themselves, *INHBA* expression led to an increase in gene signatures related to EMT transition, neuron development, neural crest cell migration, and regulation of stem cell differentiation, consistent with known functions of Activin-A and SMAD2/3 transcription factors in stimulating melanoma cell migration (16, 55, 56). While TGF-β is an established EMT inducer in various cell lines, the role of Activin-A in this process is less clear, even though both ligands share the same SMAD transcription factors for canonical signal transduction. In ovarian cancer cells, Activin-A treatment promotes EMT via canonical SMAD2/3 signaling together with SMAD4 (57), whereas in colon and breast cancer cells, it has been reported to promote EMT and cell migration or invasiveness independently of SMAD4 (7, 58). In addition, our comparison of differentially regulated gene signatures in control and Activin-A secreting tumors identified inflammatory IFN-A and IFN-γ responses as the cancer hallmark signatures that were most significantly upregulated by Activin-A in nearly all cell types, including the cancer cells themselves. We observed no corresponding increase in the transcription of either type I or II interferons aside from Th1 cells, where it also remained very modest. However, melanocytes in neonatal skin and UVB-initiated melanoma cells are protected from immune-mediated killing by IFN-γ that is secreted by CCR2^+^ macrophages (59). Thus, Activin-A may increase IFN-γ in YUMM3.3 melanoma grafts indirectly by enriching the TME for macrophages. Importantly, the striking increase in the inflammatory IFN signatures by Activin-A did not improve T cell recruitment or the expression of T cell activation markers, but instead correlates with CD8 T cell exclusion and increased tumor growth (17). We also did not observe increased recruitment of NK cells or major changes in their transcriptome, even though Activin-A has been reported to noticeably slow their proliferation and modulate their cytokine expression when added as a recombinant protein or presented by dendritic cells *in vitro* (60, 61). Chronic exposure of B16 mouse melanoma cells to IFN-γ stimulates the expression of the checkpoint receptors PD-1, CTLA-4, LAG-3, and of TIM-3 ligands, as well as the resistance to combined ICB therapies in tumor grafts (62). At the early stage of YUMM3.3 tumor growth examined here, Activin-A secretion increased the expression of the ligands *Lgals3*, *Hmgb1*, *Lgals9*, *CD274* in various cell types. This is consistent with the notion that chronic IFN-γ signaling in melanoma patients promotes immune evasion (63).

While crucial in the innate and adaptive immune responses, IFNs also have direct effects on cancer cells. For example, in prostate cancer, JAK/STAT inflammatory signaling initiates lineage dedifferentiation and outgrowth of castration-resistant organoids (64). By contrast, IFN-γ treatment of patient-derived melanoma cells for 7 days has recently been shown to inhibit growth between 20-80% in 29 out of 31 cases examined (65). Here, sustained *INHBA* expression or acute treatment of YUMM3.3 melanoma cells with recombinant Activin-A enhanced STAT1 phosphorylation by IFN-γ but interfered with cytostatic IFN-γ signaling *in vitro* without further upregulating IFN target genes. These observations indicate that Activin-A likely stimulates an inflammatory IFN signature *in vivo* by altering the TME. Several mechanisms have been described how IFN-γ can induce growth inhibition in cancer cells, including activation of ERK signaling (63, 65). Here, we found that the cytostatic effect observed in YUMM3.3 treatments was independent of apoptosis, necroptosis, ferroptosis or cell cycle arrest. Whether ERK signaling or a pseudo-EMT signature are involved remains to be determined. EMT tends to de-sensitize epithelial cancers to chemotherapies (66). However, here, upregulation of an EMT signature in YUMM3.3 cells provided no resistance to the chemotherapeutic agent carboplatin, indicating that Activin-A signaling in these cells selectively promoted the evasion of cytostatic IFN-γ signaling but not chemoresistance. IFN signaling in cancer cells is necessary for the response to immunotherapies, and loss of JAK1/2 or of IFN-inducible MHC I genes frequently accounts for therapy resistance (67–69). Furthermore, an IFN signature can positively predict responses to PD1 inhibition in several cancer types (70). However, others have found that IFN signature can be upregulated in both responders and non-responders, and that increased expression of IFN related genes can even predict poor response to ICB therapy, chemotherapy and radiation (62, 64, 71–74). Interestingly, the 18 genes of an IFN signature marking non-responders include *INHBA* and *CCL2* (72). Here, our analysis in YUMM3.3 melanoma showed that *Ccl2* was upregulated by Activin-A both in the tumor cells and in fibroblasts. Furthermore, our survey of publicly available gene expression data revealed upregulation of IFN response gene signatures and increased *STAT1* and *CGAS* expression in presence of high levels of *INHBA* transcripts in human tumors. In keeping with the dual role of IFN signaling in both inhibiting and promoting anti-tumor immunity, treatment of YUMM3.3 melanoma-bearing mice with JAK1/2 inhibitors drastically accelerated the growth of Ctrl tumors, thereby minimizing the difference compared to the growth rate of βA tumors. This corroborates the conclusion of previous reports that Activin-A promotes growth by inhibiting CD8^+^ T cell responses in several melanoma models (16, 17).

Increased IFN signaling in cancer can also be a consequence of the release of double-stranded DNA into the cytoplasm that induces the synthesis of cyclic GMP-AMP (cGAMP) by cGAMP synthase (cGAS) upstream of STING (75). Secretion of cGAMP by cancer cells stimulates STING in various immune cells, including anti-tumor NK cells (76). By contrast, in a chemically induced mouse model of squamous cell carcinoma of the skin, STING is responsible for tumor-promoting inflammation and cancer formation, acting both within epidermal cells and in bone marrow-derived immune cells (5). Furthermore, cancer cell-intrinsic STING signaling associated with an inflamed cell state has been shown to facilitate metastatic growth in preclinical models of breast cancer by activating NFκB (77), and it also mediates tumor immune evasion and immunotherapy resistance in genetically unstable BRCA1 mutant ovarian cancer by driving VEGFA-induced tumor vascularization (78). For patients receiving immunotherapies, these and possibly other tumor-promoting functions of STING are clinically highly relevant. Indeed, in clinical trials across multiple cancer types, pharmacological STING agonists that boost anti-tumor immunity showed limited efficacy when administered alone because they tend to activate immune checkpoints and tolerogenic Tregs (2). In addition, efficacy of STING agonists in B16-F10 and in the YUMM3.3-related YUMM1.7 melanoma models is limited by epigenetic inhibition of gene transcription (79, 80). Here, we found that STING expression in YUMM3.3 cells was enhanced upon treatment with IFN-γ. Furthermore, while knockdown of *Sting* did not alter YUMM3.3 cell proliferation *in vitro* or its inhibition by IFN-γ treatment, it slowed down the growth of *INHBA*-expressing tumors. Thus, Activin-A depends at least in part on tumor-promoting STING signaling in melanoma cells to accelerate the tumor growth. Overall, these observations uncover a novel interplay of melanoma cell-intrinsic STING and IFN/JAK signaling pathways that will be important to consider for future therapeutic strategies that seek to inhibit melanoma progression and immunotherapy resistance associated with paracrine Activin-A signaling.

## Data availability statement

The datasets presented in this study can be found in online repositories. The names of the repository/repositories and accession number(s) can be found below: GEO Series accession numbers GSE247229 and GSE247228.

## Ethics statement

The animal study was approved by the cantonal veterinary administration of the canton of Vaud, Chemin des Boveresses 155, 1066 Epalinges. The study was conducted in accordance with the local legislation and institutional requirements.

## Author contributions

KP: Conceptualization, Formal analysis, Investigation, Validation, Visualization, Writing – original draft, Writing – review & editing, Methodology, Project administration, Resources, Data curation. GA: Data curation, Formal analysis, Investigation, Writing – review & editing. JL: Data curation, Formal analysis, Investigation, Validation, Writing – review & editing, Methodology, Visualization. NF: Data curation, Formal analysis, Investigation, Writing – review & editing, Project administration, Supervision, Validation. CI: Supervision, Validation, Writing – review & editing, Project administration. NG: Data curation, Formal analysis, Supervision, Validation, Visualization, Writing – review & editing, Project administration. OE: Writing – review & editing, Resources. SN: Conceptualization, Formal analysis, Investigation, Visualization, Writing – review & editing, Data curation, Methodology, Resources, Software. DC: Conceptualization, Formal analysis, Funding acquisition, Investigation, Project administration, Supervision, Validation, Visualization, Writing – original draft, Writing – review & editing.
