# Supplementary material for: Inhibition of anti-tumor immunity by melanoma cell-derived Activin-A depends on STING
Source: Front Immunol. 2024 Jan 18;14:1335207. doi: 10.3389/fimmu.2023.1335207 (PMC10830842; doi:10.3389/fimmu.2023.1335207)
Supplement: Supplementary file 1 [file DataSheet_1.docx]

Supplementary Figures


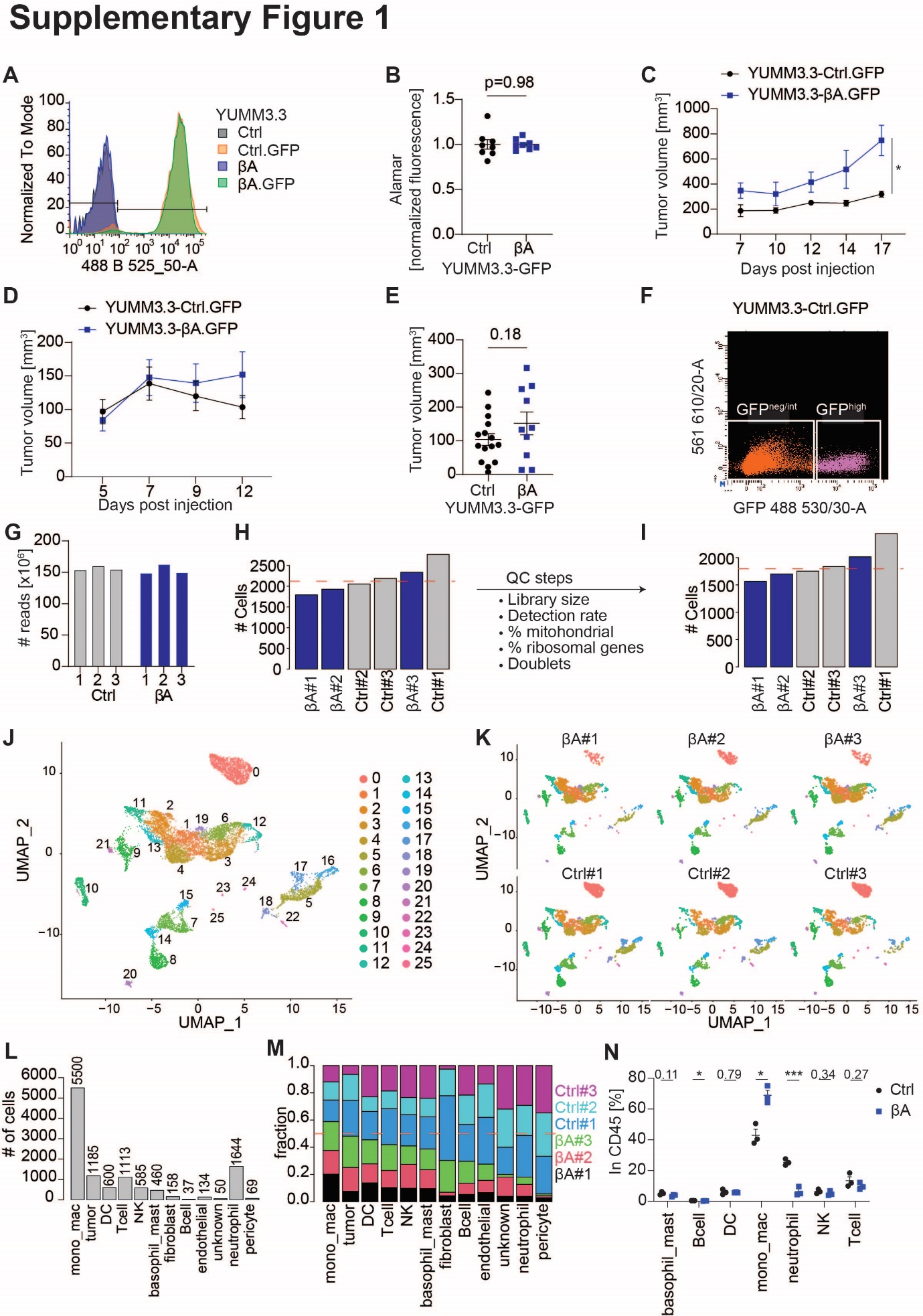


**Supplementary Figure 1.** Identification of cell types in YUMM3.3.GFP melanoma using scRNA-seq analysis. **(A)** Representative histogram of GFP expression measured by flow cytometry in YUMM3.3-Ctrl and -ꞵA cells after lentiviral transduction and in parental cells. **(B, C)** The proliferation of YUMM3.3-Ctrl. and -ꞵA.GFP cells in culture measured by **(B)** Alamar Blue assay (n = 8) and **(C)** their growth when grafted in syngeneic female C57BL/6J mice. Error bars, SEM (n = 3).; *p<0.05, Student’s t-test. **(D, E)** Tumor growth curves **(D)** of YUMM3.3-Ctrl. and -ꞵA.GFP and **(E)** tumor volumes on day 13 after tumor grafting. Error bars, SEM (n = 10 for ꞵA and 15 for Ctrl group); p value, Student’s t-test. **(F)** Representative flow cytometry gating panel for sorting GFP^hi^ tumor cells from other stromal and immune cells before the 10x Genomics analysis. **(G)** The number of gene reads in Ctrl and ꞵA triplicates identified by 10x Genomics. **(H, I)** The number of cells sequenced in each sample **(H)** before and **(I)** after the quality control assessment. **(J, K)** UMAP representation of **(J)** cell clusters in pooled Ctrl and ꞵA samples identified by unsupervised clustering, and **(K)** representation of the clusters in individual samples. **(L, M)** Total cell numbers **(L)** of cell types identified by scRNA-seq and their proportion **(M)** in YUMM3.3 tumors analyzed by 10x Genomics. **(N)** Comparison of frequencies of immune cells in Ctrl and ꞵA samples. Error bars, SEM (n = 3). *p<0.05, **p<0.01, ***p<0.001, ****p<0.0001, Student’s t-test


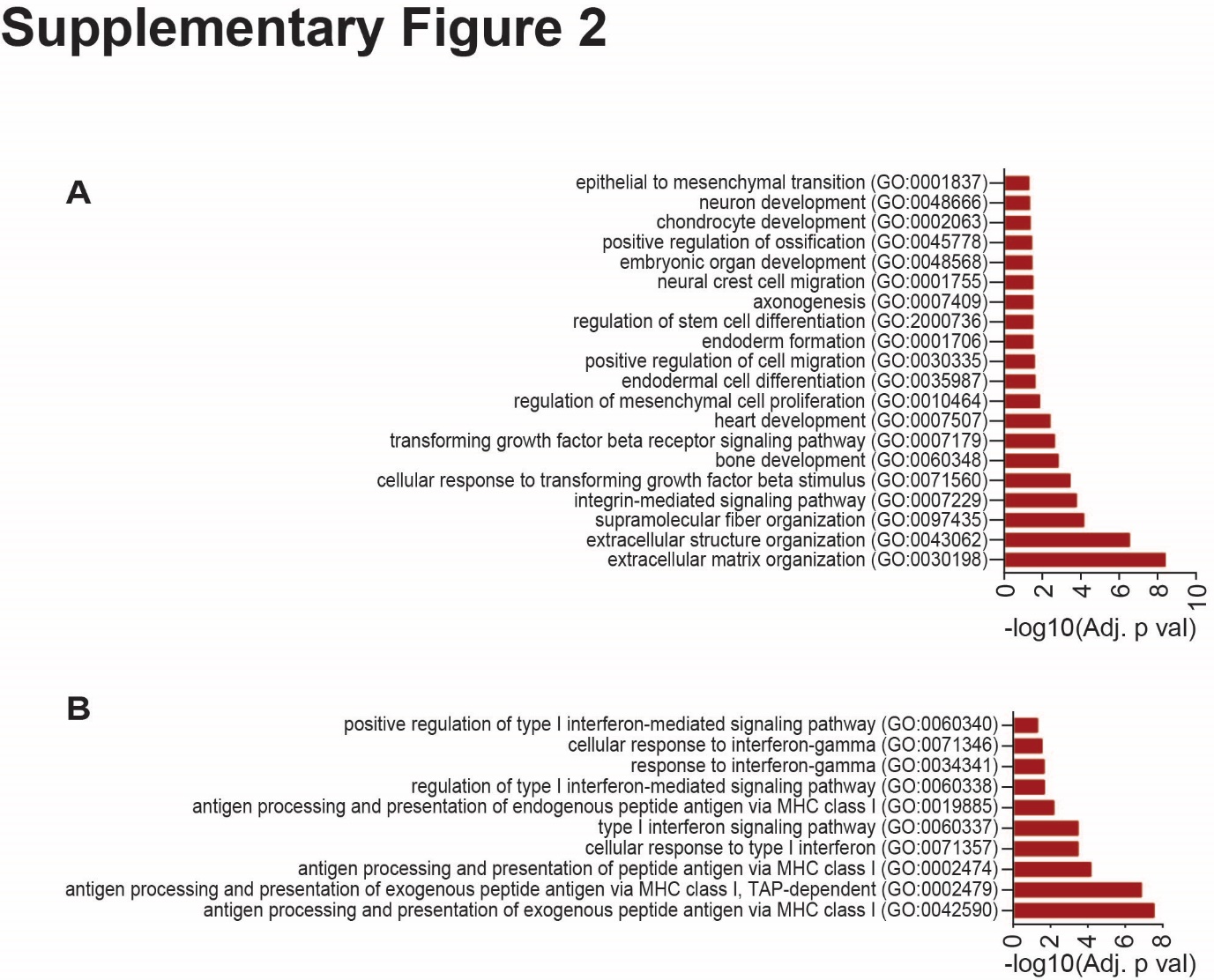


**Supplementary Figure 2.** Activin-A expression upregulates developmental programs in melanoma cells. **(A)** Gene ontology enrichment analysis of ꞵA-upregulated biological processes in YUMM3.3 tumor cells. **(B)** Gene ontology enrichment analysis of ꞵA-upregulated biological processes related to IFN signaling in YUMM3.3 tumor cells.


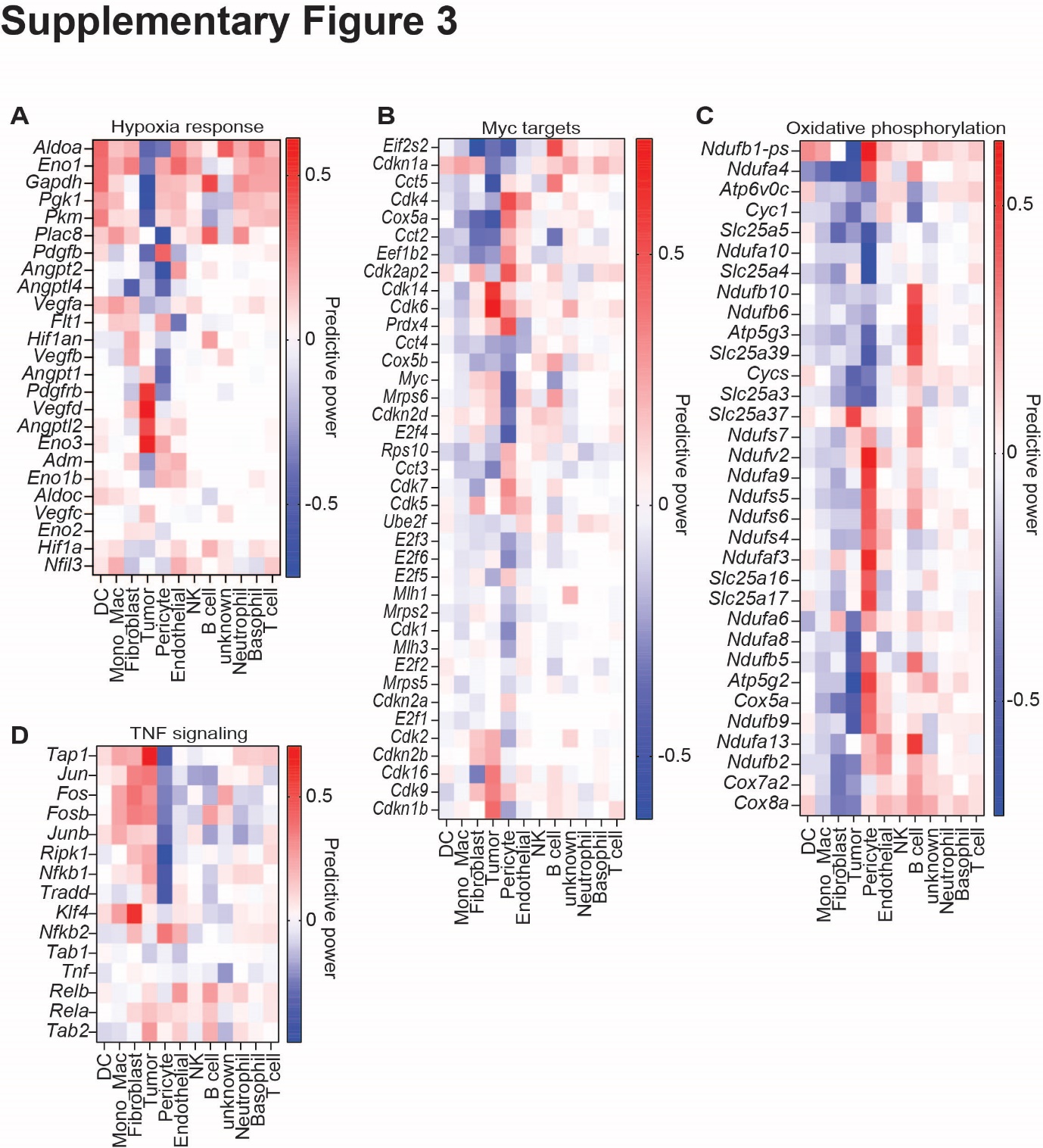


**Supplementary Figure 3.** Characterization of the leading hallmark signature alterations. **(A-D)** Heatmaps of the relative expression levels of differentially regulated genes of **(A)** hypoxia response, **(B)** Myc targets and **(C)** oxidative phosphorylation, and **(D)** TNF hallmark gene sets.


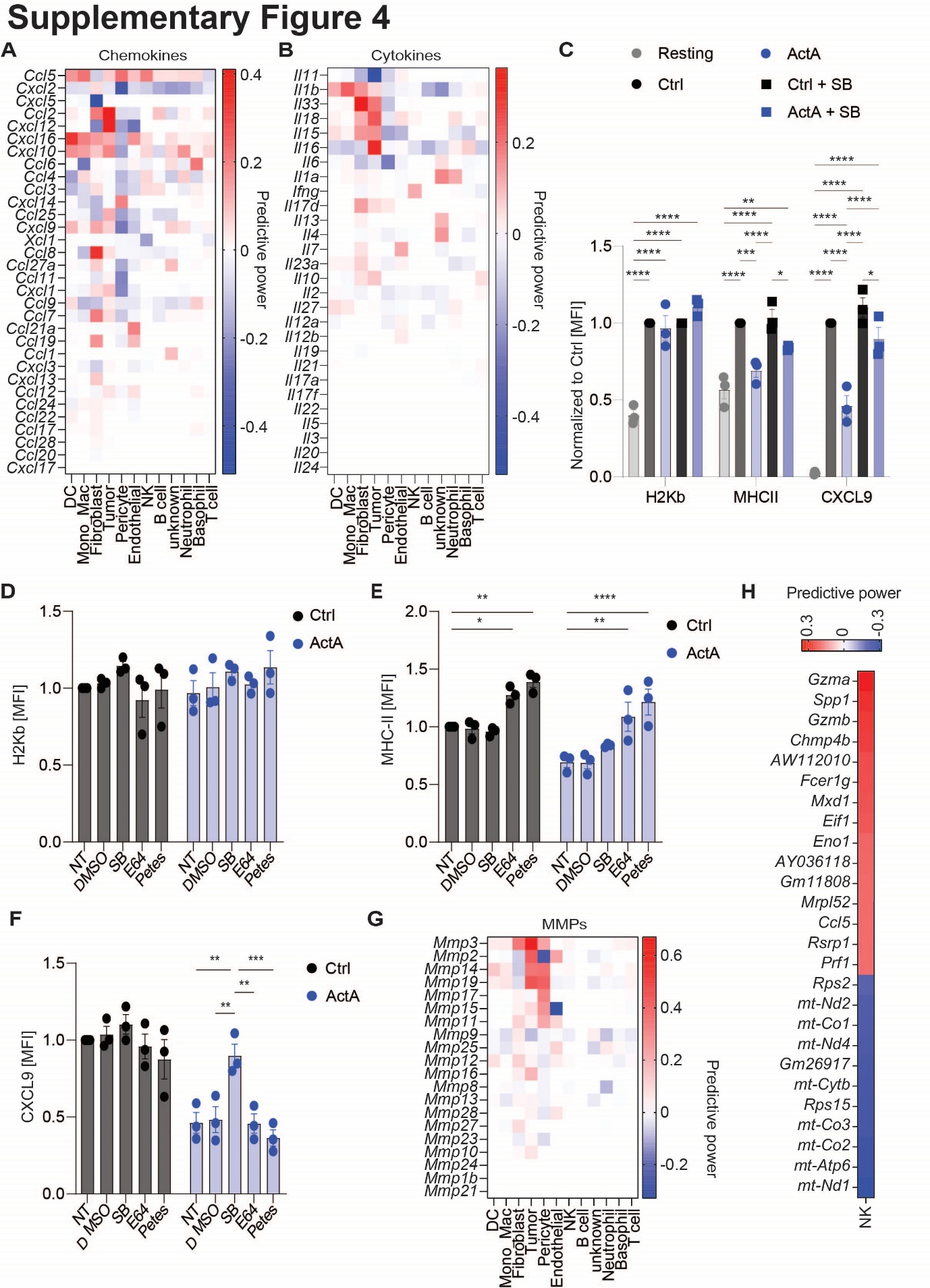


**Supplementary Figure 4.** Activin-A signaling down-regulates CXCL9 protein expression in a murine cDC1 cell line regardless of the presence of broad-specificity cysteine or serine protease inhibitors. **(A,B)** Heatmaps of the relative expression levels of **(A)** differentially regulated chemokines or **(B)** cytokines in the indicated cell types of YUMM3.3-βΑ versus -Ctrl melanoma grafts. **(C)** Intracellular CXCL9 and cell surface expression of H2Kb and MHCII by the murine cDC1 cell line measured by flow cytometry as mean fluorescence intensity (MFI) prior to activation (resting), relative to their expression after activation with 5 μg/ml LPS plus 10 ng/ml IFN-γ for 24 hrs. Where indicated, the cells were co-treated with 20 ng/ml Activin-A or with the Alk4 inhibitor SB-431542 (10 μM). **(D-F)** As in C, but levels of **(D)** H2Kb, **(E)** MHCII, and **(F)** intracellular CXCL9 after co-treatment of cells with the cysteine or serine protease inhibitors 50 μM E64 or Petes , respectively, or with empty vehicle (DMSO). Error bars, SEM (n = 3 independent experiments); *p<0.05, **p<0.01, ***p<0.001, ****p<0.0001, ordinary one-way ANOVA with Holm-Šídák correction for multiple comparisons. All data are normalized and compared to control-treated activated cDC1 control. NT= non treated **(G)** Heatmaps of the relative expression of differentially regulated MMPs in the indicated cell types of YUMM3-3-βΑ versus -Ctrl melanoma grafts. (**H**) Heatmap showing differentially regulated genes in NK cells with predictive power >0.2.


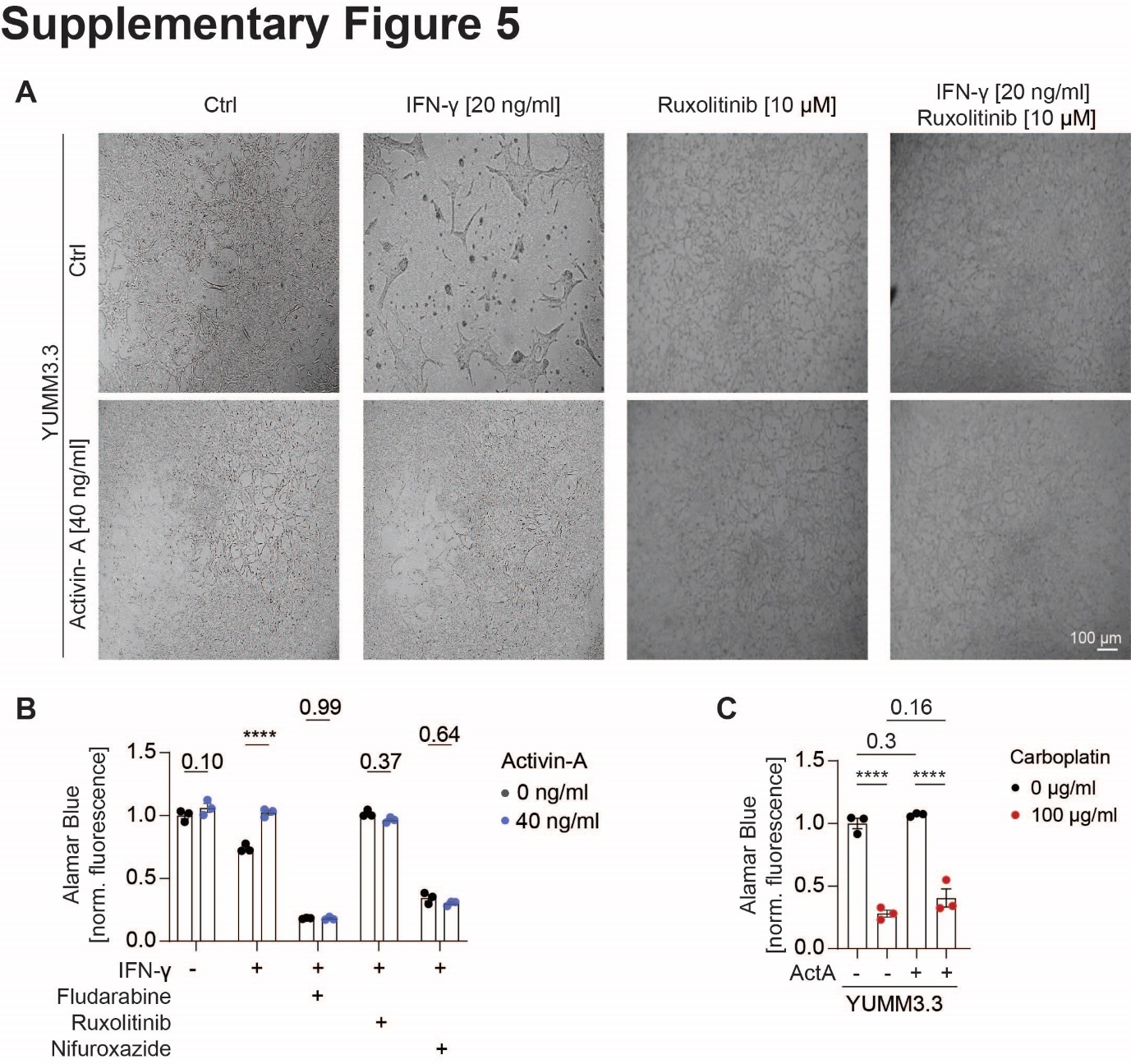


**Supplementary Figure 5.** Activin-A promotes resistance to cytostatic JAK/STAT signaling in YUMM3.3 cells. **(A)** Representative bright field images of YUMM3.3 cells treated during 48 hrs with 40 ng/ml Activin-A, 20 ng/ml IFN-γ, or 10 μM Ruxolitinib where indicated (scale bar, 100 µm). **(B)** Proliferation of YUMM3.3 cells during 48 hrs in presence of 40 ng/ml Activin-A, with or without 20 ng/ml IFN-γ. Where indicated, the cells were co-treated with an inhibitor of DNA synthesis (10 μM Fludarabine), or with the JAK inhibitor Ruxolitinib (10 µM), or with the STAT3 inhibitor Nifuroxazide (0.5 μM) . Error bars, SEM (n = 3).; ****p<0.0001, Student’s t-test. **(C)** Proliferation of YUMM3.3 cells during 48 hrs treatment with 40 ng/ml Activin-A or 100 μg Carboplatin where indicated (n = 4-5).


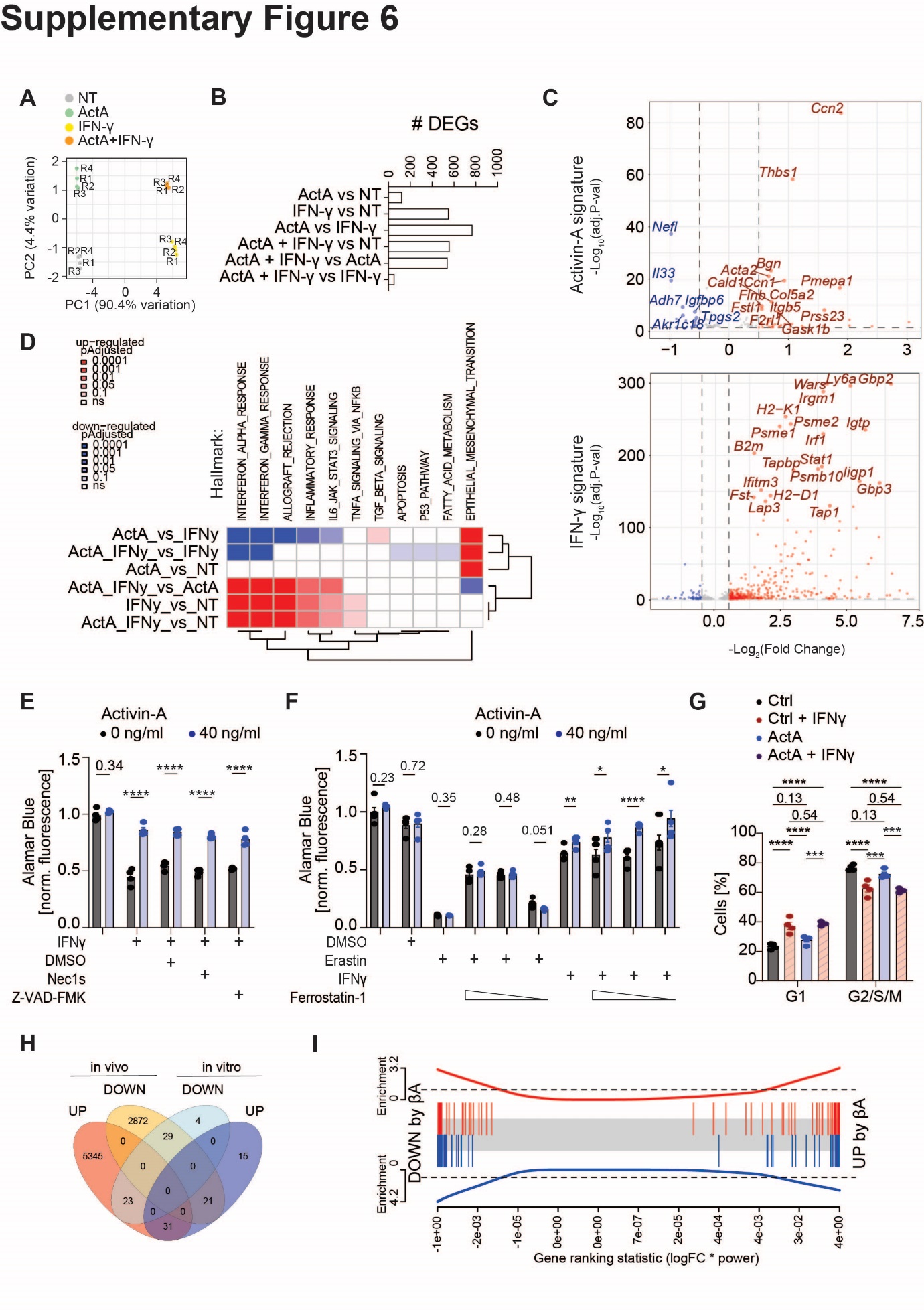


**Supplementary Figure 6.** Transient Activin-A treatment of melanoma cells downregulates IFN signaling. **(A)** PCA plot based on the top 500 most variable genes in the RNA-seq datasets of YUMM3.3 cells (grey) after treatment for 12 hrs with 20 ng/ml IFN-γ (yellow) or 50 ng/ml Activin-A (green), or both (orange). **(B)** Number of identified genes that were differentially regulated by the indicated single or combo treatments (adjusted pVal <0.05). **(C)** Significantly regulated genes in YUMM3.3 after treatment with Activin-A (top) or IFN-γ compared to the H_2_O control (bottom) as in (A, B). **(D)** Results of GSEA analysis showing changes in enrichment scores of Hallmark gene sets in YUMM3.3 cells. **(E)** Proliferation of YUMM3.3 cells during 48 hrs treatment with 20 ng/ml IFN-γ together with 40 ng/ml of Activin-A, and DMSO, 10 μM Nec1s or 50 μM Z-VAD-FMK where indicated, relative to control. Error bars, SEM (n = 4).; *p<0.05, **p<0.01, ***p<0.001, ****p<0.0001, two-way ANOVA with Holm-Šídák correction for multiple comparisons. **(F)** Proliferation of YUMM3.3 cells during 48 hrs treatment with 50 ng/ml ActA together with DMSO, 20 ng/ml IFN-γ, 5 μM Erastin or 200, 100 or 50 μM Ferrostatin-1 where indicated, relative to control . Error bars, SEM (n=8 from 2 independent experiments).; *p<0.05, **p<0.01, ***p<0.001, ****p<0.0001, Student’s t-test. **(G)** Flow cytometric quantification of the relative frequencies of YUMM3.3-Fucci cells in G1 detected by red fluorescence or in G2/S/M phases of the cell cycle detected by green fluorescence after 24 hrs treatment with or without 20 ng/ml IFN-γ together with 0 or 40 ng/ml Activin-A. Error bars, SEM (n = 3-4).; *p<0.05, **p<0.01, ***p<0.001, ****p<0.0001, two-way ANOVA with Holm-Šídák correction for multiple comparisons. **(H)** Venn diagram showing the overlap between Activin-induced DEGs in YUMM3.3 cells from scRNA-seq and BRB-seq analyses (left), with heatmaps indicating log2 fold changes of significantly (adj. pVal <0.05) up and down regulated genes (middle and right panels). **(I)** Barcodeplot indicating the location of DEGs from BRB-seq analysis on a ranked list of genes in YUMM3.3 cells from scRNAseq analysis. Up-regulated (shown in red) and down-regulated (shown in blue) DEGs from BRB-seq analysis exhibit mixed expression patterns in scRNA-seq data.


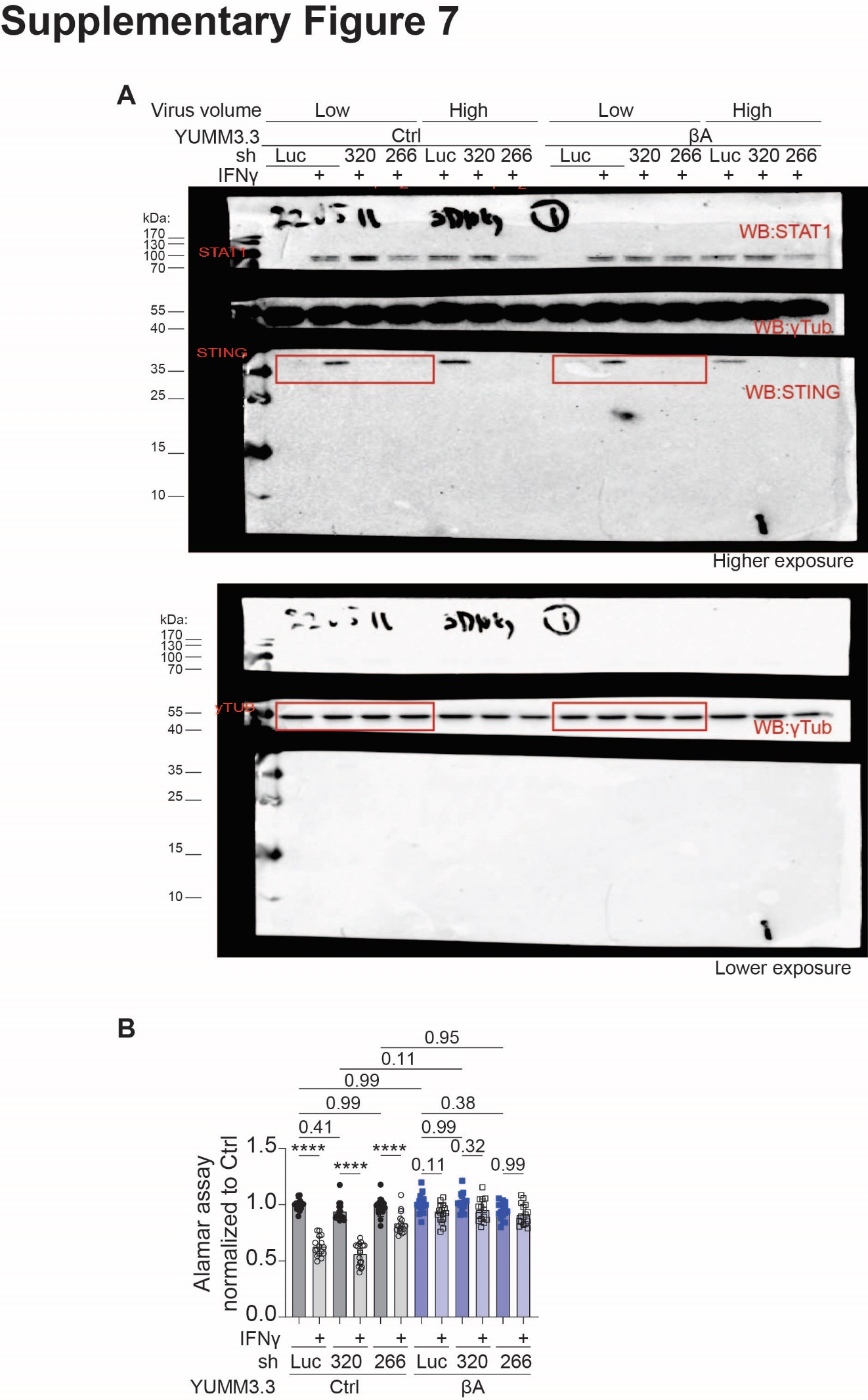


**Supplementary Figure 7.** shSting does not affect YUMM3.3 cell proliferation *in vitro*. **(A)** Uncropped versions of the Western blots in figure 7E. **(B)** Proliferation of YUMM3.3-Ctrl or -βA cells that were stably transduced with lentiviral shLuc, shSting 320, or shSting 266 expression vectors and treated during 48 hrs with 0 or 20 ng/ml Activin-A. Error bars, SEM (n=8 from 2 independent experiments); **p<0.01, ***p<0.001, ****p<0.0001, ordinary one-way ANOVA with Holm-Šídák correction for multiple comparisons.
